# Cellulose Aerogels: Synthesis, Applications, and Prospects

**DOI:** 10.3390/polym10060623

**Published:** 2018-06-06

**Authors:** Lin-Yu Long, Yun-Xuan Weng, Yu-Zhong Wang

**Affiliations:** 1 School of Materials and Mechanical Engineering, Beijing Technology& Business University, Beijing 100048, China; 15652591908@163.com; 2 Beijing Key Laboratory of Quality Evaluation Technology for Hygiene and Safety of Plastics, Beijing Technology and Business University, Beijing 100048, China; 3 Center for Degradable and Flame-Retardant Polymeric Materials, College of Chemistry, Sichuan University, Chengdu 610064, China

**Keywords:** aerogels, cellulose, sol-gel process, preparation, application

## Abstract

Due to its excellent performance, aerogel is considered to be an especially promising new material. Cellulose is a renewable and biodegradable natural polymer. Aerogel prepared using cellulose has the renewability, biocompatibility, and biodegradability of cellulose, while also having other advantages, such as low density, high porosity, and a large specific surface area. Thus, it can be applied for many purposes in the areas of adsorption and oil/water separation, thermal insulation, and biomedical applications, as well as many other fields. There are three types of cellulose aerogels: natural cellulose aerogels (nanocellulose aerogels and bacterial cellulose aerogels), regenerated cellulose aerogels, and aerogels made from cellulose derivatives. In this paper, more than 200 articles were reviewed to summarize the properties of these three types of cellulose aerogels, as well as the technologies used in their preparation, such as the sol–gel process and gel drying. In addition, the applications of different types of cellulose aerogels were also introduced.

## 1. Introduction

In today’s world, due to the increasing scarcity of oil resources and serious environmental pollution problems caused by petroleum-based polymers, biodegradable, inexpensive, and non-toxic natural polymer materials have attracted considerable attention from researchers, corporations, and governments.

Cellulose is the most abundant natural polymer on Earth. In terms of structure, it is a linear polymer formed by the linkage of d-glucose with 1,4-β-glycosidic bonds [1]. The length of its molecular chain depends on the source and extraction process of cellulose [2]. Cellulose has many properties that are different from those of petroleum-based polymers, such as biocompatibility, biodegradability, thermal stability, chemical stability, and low cost [3,4]. Its industrial applications, such as in paper, cardboard, fabric, and building materials, can be traced back thousands of years, although only the multi-layer structure and hardness of cellulose have been exploited in these materials, and they cannot meet the requirements for new materials in the 21st century in terms of functionality, durability, and homogeneity. With advancing research on the physical and chemical properties of cellulose, environmentally-friendly functional cellulose-based materials, such as cellulose fibers, cellulose films, cellulose hydrogels, cellulose aerogels, and cellulose-based composites, have been developed [5]. In particular, cellulose aerogels have the renewability, biocompatibility, and biodegradability of cellulose, while also having additional advantages such as low density, high porosity, and a large specific surface area, making it one of the most promising materials in the 21st century.

In the early 1930s, Kistler developed an aerogel for the first time by removing the liquid in a wet gel using supercritical drying [6,7]. However, the complicated multistage preparation process hindered the development of aerogels. In the past few decades, different types of aerogels, such as inorganic aerogels (i.e., SiO_2_, TiO_2_, SnO_2_, V_2_O_5_, and Al_2_O_3_) [8,9,10,11,12], synthetic polymer-based aerogels, (i.e., resorcinol–formaldehyde, polyvinylchloride, polypropylene, and polyimide) [13,14,15,16], natural macromolecule-based aerogels (i.e., alginate, protein, chitosan, and hemicellulose) [17,18,19,20,21] and carbon aerogels (i.e., carbon, carbon nanotubes, and graphene) [22,23,24], have been developed due to progress in the technologies used for the synthesis and drying of aerogels.

Aerogel is a type of special porous material with excellent physical and chemical properties, such as low density (0.003–0.500 g∙cm^−3^), high porosity (80~99.8%), large specific surface area (100~1600 m^2^/g), and adequate surface chemical activities. Technologies with the potential to be improved by aerogel include those used in the areas of optoelectronics, adsorption catalysis, sound insulation, medical materials, aerospace materials, and many other fields [25,26,27,28,29,30]. However, the mechanical properties of silica aerogels are poor [31], and the precursors of synthetic polymer-based aerogels are toxic and non-degradable. Coupled with their high cost of preparation, these factors have significantly restricted the application of aerogels.

**Figure 1:**
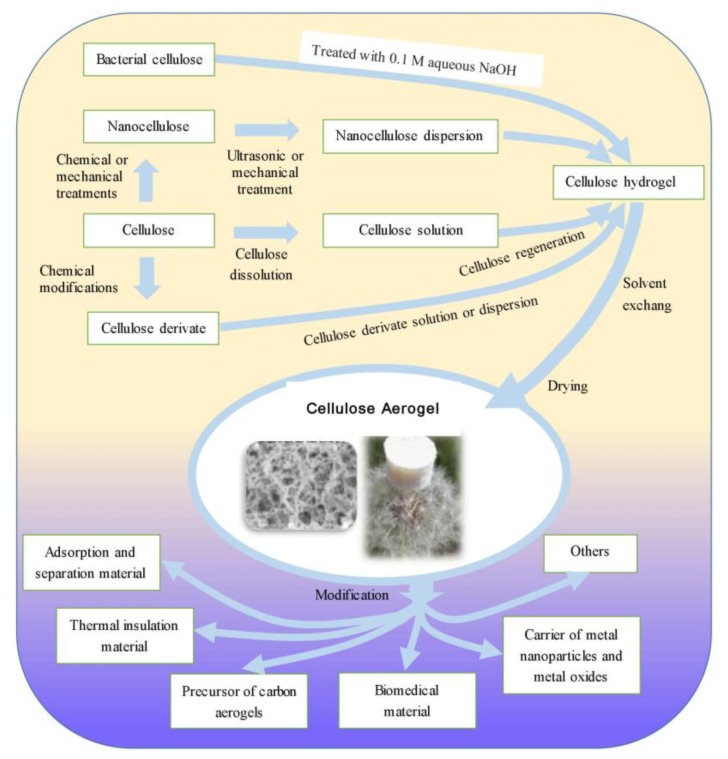
Schematic of the preparation and application of cellulose aerogels.

Cellulose aerogel is a porous solid material. Cellulose aerogel is generally prepared in three steps: dissolving/dispersing cellulose or cellulose derivatives, forming cellulose gel by the sol–gel process, and drying cellulose gel while basically retaining its 3D porous structure. Figure 1 shows the preparation process of cellulose aerogels and their applications.

The specific surface area (10–975 m^2^/g), porosity (84.0–99.9%), and density (0.0005–0.35 g∙cm^−3^) of cellulose aerogels are comparable to those of traditional silica aerogels and synthetic polymer aerogels, but cellulose aerogels have a higher compressive strength (5.2 kPa–16.67 MPa) and better biodegradability. Therefore, cellulose aerogels are a type of environmentally-friendly and multi-functional new material that has great potential in the application of adsorption and oil/water separation, heat insulation, biomedical materials, metal nanoparticle/metal oxide carriers, the preparation of carbon aerogels, and many other areas. However, as far as we know, few reviews and books have covered the preparation and application of cellulose aerogels, and only a few reviews and some book chapters have mentioned cellulose aerogels [5,32,33,34,35] or a particular type of cellulose aerogels [36,37]. Based on the recent literature regarding cellulose aerogels, this article reviewed research progress in the preparation of cellulose aerogel and its applications.

## 2. Preparation of Cellulose Aerogel

Cellulose can be extracted from a wide range of different sources [33,38,39], which mainly include plants and plant-based materials such as rice straw [40], cannabis [41], cotton [42], wood [43,44], potato tubers [45], and bagasse [46]. The performance characteristics of cellulose, such as its molecular chain length (degree of polymerization, DP), size, degree of crystallinity, and thermal stability [47,48], are determined by the species of plant from which it is derived, as well as the extraction processes used in its production, including the pretreatment, post-treatment, and disintegration processes; therefore, the structure and performance of cellulose aerogels are influenced by the plant source from which their cellulose is derived [49,50]. Cellulose can also be synthesized by the static culturing of *Acetobacter xylinum* and other bacteria. However, although the chemical structure of cellulose obtained from bacteria culture is the same as that of plant cellulose, the former has a higher degree of crystallinity (>80%). In addition, bacterial cellulose does not contain impurities such as lignin and hemicellulose, and its physical and biological characteristics are also better than those of plant cellulose [51,52,53]. Furthermore, cellulose with a low molecular weight can be synthesized in vitro through cellulase catalysis or ring-opening polymerization [54]. To the best of our knowledge, no available literature has discussed cellulose aerogels prepared from synthetic cellulose, although synthetic cellulose typically has high purity and a short production cycle, and its molecular weight is easily controlled [54]. These characteristics of synthetic cellulose make it an ideal raw material for the preparation of cellulose aerogels. It is worth mentioning that each of the glucose units in the cellulose chain has three hydroxyl groups (two secondary alcohols located in C2 and C3, respectively, and one primary alcohol located in C6) with high chemical reactivity. Therefore, cellulose derivatives such as carboxymethylcellulose, cellulose ester, and cellulose ether can be obtained by grafting, sulfonation and 2,2,6,6-tetramethylpiperidine-1-oxyl (TEMPO)-mediated oxidation [55,56]. At present, there are a large number of reviews on the structure, properties, and applications of cellulose and its derivatives [57,58,59,60]. In order to avoid redundancy, we will not repeat the contents of those reports in this article.

Cellulose and its derivatives can enhance the mechanical properties and moisture affinity of aerogel materials [61,62,63]. In addition, the following advantages can be obtained by using cellulose as the precursor for the preparation of aerogels. (1) First, the reserve of cellulose raw material is inexhaustible and renewable; (2) Second, the cellulose chain is rich in hydroxyl groups, so no cross-linking agent is needed in the aerogel preparation process. A stable three-dimensional (3D) network structure can be obtained by intramolecular and intermolecular physical cross-linking of hydrogen bonds, thus making the aerogel preparation process quite simple; (3) Third, the chemical modification of cellulose to improve the mechanical strength and structural characteristics of cellulose aerogels is relatively easy to accomplish.

The preparation method and structural properties of cellulose aerogels are largely dependent on the performance of cellulose and its concentration. Therefore, cellulose aerogels are divided into three categories based on their raw materials: natural cellulose aerogels (nanocellulose aerogels, bacterial cellulose aerogels), regenerated cellulose aerogels, and cellulose derivate aerogels. The preparation of these three types of aerogels and differences in their performance are discussed below.

### 2.1. Sol–Gel Process

In a sol, colloidal particles with diameters ranging from 1 nm to 1000 nm are dispersed in a liquid. A gel consists of a sponge-like, three-dimensional solid network whose clusters are filled with another substance (usually a liquid) [64]. The sol–gel reaction is a process in which the material transforms from the liquid sol phase to the solid gel phase. The sol–gel reaction is the most critical step in the formation of a 3D porous network structure in an aerogel. At present, almost all of the aerogels are obtained by a wet chemical synthesis: the sol–gel method [64,65,66].

The cellulose solution or suspension can lead to a gel by agglomeration of polymers or by a phase separation process when coagulative regeneration is used. The exchange of solvent with non-solvent (regeneration) leads to a desolvation of the cellulose molecules and to the supposed reformation of the intramolecular and intermolecular hydrogen bonds. The gelation of cellulose from, for instance, *N*-methylmorpholine-*N*-oxide (NMMO) solutions using water as the coagulation system results from a phase separation process that forms polymer-rich and polymer-poor phases [67]. Typically, two mechanisms of phase separation can take place during liquid–liquid demixing of polymer solutions; either nucleation/growth (i.e., the nuclei of one phase grows in the mixture), or spinodal decomposition (i.e., a periodic variation of concentration leads to the final phase separation) [68].

In general, by adding chemical crosslinking agents such as epichlorohydrin (ECH) and *N*,*N*′-methylenebisacrylamide (MBA) to the liquid sol or changing physical conditions (temperature, pH, ultrasonic treatment, etc.), colloidal particle aggregation can be induced to form a 3D interconnected network structure, thus converting the material into a solid gel [69,70].

Physical gels are cross-linked by physical interactions such as van der Waals forces, hydrogen bonds, hydrophobic or electronic associations, and chain entanglements. Usually, the gelation speed of physical gels mainly relies on the concentration of cellulose solution or dispersion and the temperature. The stable structure and effective swelling of cellulose gels are usually achieved by the use of chemical cross-linkers, which can form covalent bonds between polymer chains during gelation. In contrast to the covalent bond in chemical gels, the binding energy of cross-links in physical gels is of the order of thermal energy, so that the network junctions can be created and destroyed by the thermal motion of polymers, thus leading to unique properties of physical gels. In addition, the degree and speed of phase separation depend on the species or concentration of the anti-solvent and temperature.

In general, the gelation speed achieved by chemical cross-linking is faster than that achieved by physical gelation, and a more stable gel structure can be formed in this way. In addition, electrolytes such as calcium chloride can change the charge distribution in the solution and promote the physical gelation process [71,72]. Graphene oxide (GO) can form hydrogen bonds with cellulose, so it can also accelerate the process of physical gelation [73].

The formation of a gel can be determined by the following methods: (1) the gel will not flow when the mold is tilted by 70° or inverted; (2) the storage modulus (G′) is equal to the loss modulus (G″) [67,74].

The sol–gel process varies based on the particular type of cellulose aerogel desired. For example, because the molecular chains of cellulose derivatives have a reduced number of hydroxyl groups, a cross-linking agent is generally needed to obtain a stable gel structure. Regenerated cellulose gel is prepared by the regeneration of cellulose solutions, whereas nanocellulose gel is made from a nanocellulose suspension.

#### 2.1.1. Natural Cellulose Aerogels

Since there is a broad range of different hydrogen bond connection networks and changes of molecular direction in cellulose, cellulose is associated with a variety of different crystalline structures, which depend on the source of cellulose, extraction method, and post-treatment processes. There are six known cellulose crystal structures: I, II, III_1_, III_2_, IV_1_, and IV_2_. The crystalline structure of natural cellulose is cellulose I, which has two sub-forms: I_α_ and I_β_ [75,76]. The crystalline structure of bacterial cellulose is usually cellulose I_α_, whereas plant cellulose can have both I_α_ and I_β_ structures [3]. Table 1 summarizes the current literature related to natural cellulose aerogels, including nanocellulose aerogels and bacterial cellulose aerogels. This table describes the drying methods, density, porosity, specific surface area, and modulus of natural cellulose aerogels.

##### Nanocellulose Aerogels

Nanocellulose fibers have a diameter of less than 100 nm [94,95] and are separated from pure cellulose using mechanical [45,96,97,98,99,100] or chemical [101,102] approaches. According to differences in separation methods, nanocellulose can be divided into two categories: (i) cellulose nanocrystals (CNC) or cellulose whiskers, and (ii) cellulose nanofibers (CNF), which are also known as nanofibrillar cellulose (NFC) or microfibrillated cellulose (MFC) [32]. Detailed information regarding differences in the extraction methods and the average size for the various types of nanocellulose can be found in Klemm’s review [60].

Nanocellulose aerogels are prepared by dispersing nanocellulose in water using ultrasonic or mechanical methods, followed by subsequent drying with or without solvent exchange. In comparison with other types of cellulose, nanocellulose has a higher degree of crystallinity and a larger aspect ratio. Therefore, compared with other cellulose aerogels, the shrinkage rate of nanocellulose aerogels is very low (<7%), and their modulus can be as high as 5.93 MPa [89].

The skeletal structures of nanocellulose aerogels consist of randomly connected bundled nanofibers, thus resulting in no optical transparency and no linear elasticity, and much lower surface areas than expected. In addition, large amounts of chemical reagents and a significant amount of energy are required during the chemical separation of nanocellulose, thus increasing its cost and hindering the development of nanocellulose aerogels.

##### Bacterial Cellulose Aerogels

Bacterial cellulose is collected from static bacterial cultures and has a natural 3D network gel structure [51]. After the removal of bacteria and other impurities and subsequent drying, cellulose aerogels can be obtained. Although the chemical structure of bacterial cellulose is similar to that of plant cellulose [103], bacterial cellulose does not contain organic impurities such as lignin and hemicellulose, and thus has certain advantages such as high purity, a high degree of polymerization, and a high degree of crystallinity [104]. Therefore, bacterial cellulose aerogels are associated with the highest modulus among cellulose aerogels [86], as well as high porosity and a high specific surface area.

On the other hand, the production of bacterial cellulose is challenged by a long production cycle (30 d), low yield, and high cost, thus reducing its attraction among academic researchers.

#### 2.1.2. Regenerated Cellulose Aerogels

Among all types of cellulose, cellulose I is not associated with the most stable crystalline structure. In fact, it is possible to obtain cellulose II, which is more stable thermodynamically, by dissolution and regeneration or mercerization treatment [75,76]. Regenerated cellulose aerogels are currently studied very extensively. The preparation of regenerated cellulose aerogels has four main steps: cellulose dissolution, cellulose regeneration, solvent exchange, and drying. Table 2 summarizes the current literature on regenerated cellulose aerogels and describes cellulose solvents, drying methods, and the properties of regenerated cellulose aerogels.

Due to the complex intramolecular and intermolecular hydrogen bond network in cellulose, it is not soluble in water and other typical organic solvents such as ethanol [135]. On the other hand, cellulose macromolecules are amphiphilic. Therefore, cellulose solvents must eliminate hydrogen bond networks and hydrophobic interactions [136]. Conventional cellulose solvents such as carbon disulfide are environmental pollutants, but environmentally-friendly cellulose solvents such as alkali (NaOH or LiOH) solution systems (alkali/water [137], alkali/water/urea or thiourea, and polyethylene glycol (PEG) [138,139,140]), LiCl/DMSO [141,142], LiCl/dimethylacetamide (DMAc) [143,144], and ionic liquids (ILs) [145] are currently used in the preparation of regenerated cellulose aerogels (see Table 2). Cellulose solvent systems can affect the performance of regenerated cellulose [146]. Therefore, aerogels prepared using different cellulose solvent systems may have different properties [105], and the selection of cellulose solvent systems during the preparation of regenerated cellulose aerogels is very important.

The preparation of regenerated cellulose aerogels requires dissolution–regeneration and multiple steps of solvent exchange, which are time-consuming. The rate of shrinkage of regenerated cellulose aerogels is generally >30%. Thus, regenerated cellulose aerogels are denser than natural cellulose aerogels and have a larger mean pore size. On the other hand, since the production process of regenerated cellulose aerogels is simple and low-cost, it has been studied most extensively.

#### 2.1.3. Cellulose Derivative Aerogels

Chemical modifications that can change the physical and chemical properties of cellulose are an important way to functionalize cellulose aerogels. Some cellulose derivatives are soluble in water and typical organic solvents. For example, carboxymethylcellulose (CMC) and hydroxypropyl methylcellulose (HPMC) are soluble in water, triacetyl cellulose (TAC) is soluble in dioxane/isopropanol, ethyl cellulose (EC) is soluble in dichloromethane, and cellulose acetate (CA) is soluble in acetone. Since acetone and some other organic solvents are soluble in ScCO_2_, the time-consuming solvent exchange process can be omitted [147], thus improving the efficiency of aerogel synthesis. On the other hand, because the molecular chains of cellulose derivatives have a reduced number of hydroxyl groups, a cross-linking agent is generally required during the gelation of the solution [148,149]. Due to the uneven distribution of the substituent groups, the effect of different degrees of substitution of cellulose derivatives on the performance of cellulose aerogels remains unclear. With respect to published research results, the degree of substitution has shown no significant effect on the density or compressive modulus of cellulose aerogels [150,151], but a high degree of substitution can reduce the hygroscopicity [151].

The process of preparing nanocellulose derivative aerogels is the same as that of nanocellulose aerogels (Section “Nanocellulose Aerogels”). At present, the most common types of nanocellulose derivative aerogels are nanocellulose aerogels oxidized by 2,2,6,6-tetramethylpiperidine-1-oxyl radicals (TEMPO) and nanocellulose aerogels with functionalized surfaces. TEMPO can selectively oxidize the primary alcohols on the molecular chain of cellulose and introduce negatively charged groups (such as carboxyl groups) into cellulose fibers to increase the separation of nanocellulose and produce more homogeneous suspensions [32]. Therefore, compared with nanocellulose aerogels, TEMPO–nanocellulose aerogels have a higher specific surface area and greater density. Surface-functionalized nanocellulose includes maleic acid-grafted CNF (CNF-MA) [152], bifunctional (aldehyde and carboxyl) nanocellulose (BMCC), and cross-linked carboxymethyl chitosan (CMCT) [153], as shown in Table 3. Table 3 summarizes the literature related to cellulose derivate aerogels and nanocellulose derivate aerogels, and lists their respective properties.

### 2.2. Gel Drying

Drying is the most critical step in the preparation of aerogels. The morphology of cellulose aerogels strongly depends on the method of drying. When conventional drying methods are used, the capillary pressure induced by the bending of the air–liquid interface can cause the gel pore structure to collapse and crack. Therefore, supercritical drying (using i.e., alcohol, acetone, or CO_2_) and vacuum freeze-drying are generally utilized in current methods of cellulose aerogel preparation. Freeze-drying is a sublimation of the solid, usually frozen water, from the pores of a wet precursor. In supercritical (sc) conditions, the liquid/gas surface tension is zero, because there is no longer liquid/gas meniscus. Aerogels prepared by drying with scCO_2_ usually present a cauliflower-like arrangement of cellulose: an agglomeration of small shaggy beads. However, freeze-drying leads to a sheet-like cellulose network with large and interconnected pores that are several micrometers in diameter due to ice growth during water freezing [165].

#### 2.2.1. Supercritical Carbon Dioxide Drying

Since CO_2_ has a suitable critical point (304 K, 7.4 MPa) and the advantages of low cost and high safety, it is a kind of fluid that is most commonly used for the process of drying cellulose aerogels. Supercritical drying involves a two-way mass transfer of scCO_2_ and gel solvent to and from the pores of the wet gel [17]. Firstly, the drying is predominantly influenced by a high dissolution of scCO_2_ in the liquid gel solvent, leading to an expanded liquid and the spillage of the excess liquid volume out of the gel network. Secondly, the CO_2_ content in the pore gel liquid increases with time until supercritical conditions are attained for the fluid mixture in the pores, without any previous intermediate vapor–liquid transition. Finally, the presence of supercritical fluid mixtures in the pores with no liquid phases leads to the absence of surface tension, thus avoiding the pore collapse phenomenon in the gel structure (i.e., changes in the macroscopic level) during solvent elimination [17].

The water that has high surface tension may damage the fragile and highly porous structure of the cellulose network, which is initially formed after the drying process. The reasons why it happens are the formation of inward forces alongside the capillary walls adjacent to the solvent menisci and arise from differences of the specific energies of the solid–liquid and liquid–gas phase transitions. So, it is necessary to completely replace the water that has high surface tension [79]. For example, when regenerated cellulose aerogels are prepared in an NMMO solvent system, the cellulose gel should be re-primed with water, followed by ethanol and acetone exchange or acetone exchange alone [105,118]. When an ionic liquid is used as the solvent system, the cellulose gel must be re-primed with water first, followed by multiple acetone exchange [166]. Natural cellulose aerogels are generally subject to ethanol exchange [71,82].

The residue of cellulose solvents can reduce the drying performance [106]. In addition, the surface tension of different liquids and shaking during the process of re-priming and solvent exchange may damage the gel structure of cellulose [106,111]. The solvent exchange process is very slow, and generally takes 2–3 d. In summary, supercritical drying by scCO_2_ can help to avoid damage to the gel 3D network caused by capillary pressure inside the pores, which allows the production of aerogel materials with a more uniform structure. However, this process involves expensive equipment, because it needs a high-pressure vessel.

#### 2.2.2. Vacuum Freeze-Drying

Vacuum freeze-drying is a simple and environment-friendly drying approach to produce cellulose aerogels. During the freeze-drying process, the gel is first frozen at a temperature below the freezing point of the liquid medium (usually water), after which the liquid is mainly eliminated by sublimation, which is a key factor in preventing structural collapse and limiting shrinkage. Therefore, liquid crystallization and growth behavior, which depend on the cooling rate and temperature, play an important role in the pore structure (pore morphology and pore distribution) of porous aerogels. The rate of sublimation is also influenced by many factors (i.e., the concentration of cellulose, the size and shape of gel, temperature), and is often slow.

Aerogels made from nanocellulose and its derivatives are generally freeze-dried, but the self-agglomeration of nanocellulose can lower their specific surface area. *tert*-butyl alcohol has low interfacial tension and contains only one hydroxyl group that can form a hydrogen bond with the hydroxyl or carboxyl group on the surface of nanocellulose and its derivatives. At the same time, the steric hindrance induced by a large number of butyl groups can prevent the agglomeration of nanocellulose. Therefore, the use of *tert*-butyl alcohol in solvent exchange can better protect the gel structure of nanocellulose and its derivatives as compared to water, thus more effectively preventing the cellulose aerogel structure from collapsing [72,108,157,167].

When thermal conductivity is increased using liquid nitrogen or liquid propane, cellulose gel can be rapidly cooled, which further inhibits the agglomeration of cellulose and the growth of ice crystal, thus increasing aerogel porosity. Zhang et al. studied three cooling rates provided by liquid nitrogen (−196 °C, 30 min), an ultralow temperature freezer (−80 °C, 12 h), and a conventional refrigerator (−20 °C, 24 h). They found that liquid nitrogen induced the rapid formation of ice crystals, which effectively inhibited the self-agglomeration of cellulose and produced a more uniform and smooth surface structure [83]. Anti-freezing agents [168] and spray freeze-drying techniques [84,169] also rely on an accelerated freezing rate to produce aerogels with a uniform structure. However, there are similar freezing rate and local temperature gradients ahead of the moving solid–liquid interface, such as when freeze-drying thin samples in a fridge while also cooling a big sample.

The drying technology that is used to produce a particular type of cellulose aerogel greatly influences its specific surface area and pore size distribution [71,119]. Usually, due to the growth of ice crystals and the high interfacial tension of water, freeze-drying produces cracks in the aerogel material. Other drawbacks of freeze-drying include its long processing times and high electric energy consumption. On the other hand, drying by scCO_2_ can better protect the gel structure of cellulose and produce aerogels with a low rate of shrinkage, a smaller pore size, and a higher specific surface area [82,110,116,170].

## 3. Applications of Cellulose-Based Aerogels

Due to cellulose’s high chemical reactivity, the large number of different derivatives with different functions, flexible preparation process, and numerous methods of modification, cellulose aerogels are generally multi-functional. There are three main ways to modify cellulose aerogels:

(1) Add other components in the solution/suspension of cellulose. For example, Gawryla et al. added a montmorillonite suspension dropwise into a suspension of nanocellulose and subjected the mixture to freeze-drying after it was mixed evenly. Using this method, they obtained an aerogel with a nanoscale wattle and daub structure and high modulus [77].
(2) Coat or apply other substances, such as SiO_2_, onto the surface of cellulose gel using a sol–gel method (see Section 3.2).
(3) There are many techniques available to achieve the surface modification of cellulose aerogels, including modification by a silane coupling agent and atomic layer deposition.

Cellulose aerogels are ultra-light 3D porous materials. Currently, they are mainly used in adsorption and separation, insulation, and biomedical applications. They are also used in the preparation of carbon aerogels and to carry metal nanoparticles/metal oxides.

### 3.1. Adsorption and Separation Materials

Frequent oil spill incidents and the discharge of oil-containing industrial wastewater during crude oil extraction and transport can cause significant economic losses and damage to aquatic ecological environments. Traditional adsorbent materials, including polypropylene (PP), zeolite, and activated carbon, are often used in the treatment of these accidents, but they suffer from disadvantages such as poor reusability, insufficiently selective oil adsorption capacity, and a lack of biodegradability [35,171,172,173]. Although natural adsorption materials made of kapok fiber, bagasse, raw cotton fiber, and coconut shell [174,175,176,177] have appropriate adsorption properties and biodegradability, they also have shortcomings such as a low selective adsorption capacity, weak buoyancy, and poor water resistance [35,173,178].

Therefore, cellulose aerogels, with their porous structure, large specific surface area, and light density are highly adsorptive for water, oil, and organic solvents [121,179,180]. The adsorption capacity of cellulose aerogels is one order of magnitude higher than that of natural adsorbents and several times that of commercial PP adsorbents. In addition, cellulose aerogels can adsorb dyes such as Congo red and methylene blue from water [151,153,164,181,182], and are biodegradable. Therefore, cellulose aerogels have received increasing attention in recent years.

The oil adsorption performance of cellulose aerogels is related to the density, viscosity, and surface tension of oily liquids, and is also dependent on capillary effects, van der Waals forces, and hydrophobic interactions, as well as density and morphological characteristics of cellulose aerogels such as their surface wettability, total pore volume, and pore structure. The adsorption performance of cellulose aerogels is affected by liquid viscosity in two ways. First, a liquid of lower viscosity tends to penetrate into the porous network of aerogels, but this property limits the adhesion between the liquid and the pore walls [125,126,183]. Cellulose aerogels with low density, high porosity, and a large pore volume tend to have a large internal free volume and high adsorption capacity [183,127,128].

On the other hand, there are a large number of hydroxyl groups on the surface of cellulose aerogels that are amphiphilic and associated with poor oil/water selective adsorption capacity [85,162]. By increasing the surface roughness of cellulose aerogels or introducing substances with low surface energy, the hydrophobicity and lipophilicity of cellulose aerogels can be improved, thus significantly enhancing the oil/water selective adsorption capacity of the aerogels. The surface modification methods and post-modification adsorption performance of different cellulose aerogels are shown in Table 4.

As shown in Table 4, commonly used methods for hydrophobizing cellulose aerogels include chemical vapor deposition (CVD) using coupling agents such as trimethylchlorosilane (TMCS), methyltrimethoxysilane (MTMS), methyltrichlorosilane (MTCS), and octadecyltrimethoxysilane (OTMS), *n*-dodecyltriethoxysilane (DDTS), atomic layer deposition, cold plasma treatment, hydrophobic modification using isocyanate cross-linking [160], surface fluorination [87,88] or esterification [186,187], and alkyl ketene dimer (AKD) modification [188]. After hydrophobic modification of cellulose aerogels, their water contact angle (WCA) is usually >135°, and their adsorption performance regarding oil and organic solvents is generally in the range of 10–400 g∙g^−1^; these characteristics indicate a performance equivalent to that of carbon aerogels and polymer-based aerogels [189,190].

Oil and water separation can also be achieved by creating a hydrophilic rough surface on the aerogels. Peng et al. have prepared a superhydrophilic cellulose aerogel by mixing cellulose and chitosan solutions. After immersing the aerogel into water, its rough surface formed a thin layer of water film, and thus possessed an ultraoleophobic capability underwater. The aerogel was used to effectively separate oil–water mixtures through filtration [191], although its reusability was limited.

Modification by a silane coupling agent is currently the most important way to hydrophobically modify cellulose aerogels. Aerogels modified in this way have excellent selective oil adsorption capacity. However, the cost of the silane coupling agent is high. In addition, the porous structure of cellulose aerogels is fragile. After the adsorption–desorption cycle, the internal structure of the cellulose aerogel is damaged, which decreases its adsorption performance, thus limiting its actual applications.

### 3.2. Thermal Insulation Material

Thermal conduction by aerogels is generally categorized as solid-state thermal conduction, gas-phase thermal conduction in an open pore structure, and radiation thermal conduction. According to the Knudsen effect, when the pore size in a porous material is close to the average free path (70 nm when ventilated) of the gas, the thermal conductivity of the material will be reduced because the pores will restrict gas movement and inhibit convection.

The thermal conductivity of mesoporous cellulose aerogels is mainly determined by their solid state thermal conduction and gas phase thermal conduction, which are in turn closely related to the aerogel density (determined by the initial concentration of cellulose), pore size distribution, and surface structures. Table 5 describes the density, pore size, and thermal conductivity of cellulose aerogels and corresponding measurement techniques. As shown in Table 5 [192], the thermal conductivity of cellulose aerogels is between 0.018–0.075 W∙m^−1^∙K^−1^ and is typically less than 0.045 W∙m^−1^∙K^−1^, which is between that of modified silica aerogels (0.041 W∙m^−1^∙K^−1^) [31] and common commercial insulating materials such as polyurethane foams (0.026 W∙m^−1^∙K^−1^), mineral wool (0.03–0.05 W∙m^−1^∙K^−1^), glass fiber (0.04 W∙m^−1^∙K^−1^), and polypropylene foam (0.030 W∙m^−1^∙K^−1^) [64,193,194,195].

In the porous structure of regenerated cellulose aerogels, the proportion of large pores is higher than that of other types of cellulose aerogels. Since large pores enhance gas transmission, they also increase thermal conductivity. Karakagli et al. prepared bulk cellulose aerogels with a pore size of 10–100 nm by dissolving microcrystalline cellulose (MCC) in an aqueous calcium thiocyanate solution and subjecting the mixture to ethanol exchange and supercritical drying. Due to the presence of large pores in the gel structure and test errors, its thermal conductivity was as high as 0.04 W∙m^−1^∙K^−1^ [107]. They also found that the thermal conductivity of the aerogel was proportional to the MCC concentration [107], which was consistent with the findings of the Seantier group [169]. However, Lu et al. argued that the structure of 3% cellulose aerogels was better than the structure of 2% cellulose aerogels. Since the 3% cellulose aerogels had lower density and higher porosity in comparison with the 2% cellulose aerogels, their thermal conductivity (0.029 W∙m^−1^∙K^−1^) was lower [109]. The Seantier group has reduced some of the large pores (120–300 μm) in bleached cellulose fibers (BCF) to the nanoscale by adding CNC, thereby reducing gas phase thermal conductivity. The overall thermal conductivity of the modified aerogel was decreased from 0.028 W∙m^−1^∙K^−1^ to 0.023 W∙m^−1^∙K^−1^ [62]. In addition, silanization can also reduce the average pore size in cellulose aerogels and thus decrease their thermal conductivity [124,196].

SiO_2_ aerogels are a common type of ultra-adiabatic material, but their poor mechanical strength greatly limits their practical applications. Cellulose–SiO_2_ aerogels can be synthesized using the sol–gel method, direct embedment, or a forced flow impregnation process. These methods can be used to produce ultra-adiabatic aerogels with good mechanical strength, low cost, and low hydrophilicity. SiO_2_ can be embedded in the large pores of cellulose aerogels to reduce their average pore size and enhance the Knudsen effect, thereby reducing the gas phase thermal conductivity [132,133]. In addition, Fu et al. found that the silica particles attached to the nanocellulosic scaffold could promote the thermal stability of the cellulose matrix [197]. GO can also enhance the thermal stabilities of cellulose, because of the formation of an extensive H-bonded network between the GO and the cellulose [198]. However, as the content of SiO_2_ increases, the specific surface area and density of the aerogels increase, and the aerogels may even rupture. In addition, their solid-state thermal conductivity also increases, thus increasing the thermal conductivity of the composite aerogels [86,89,131,192].

Cellulose aerogels have low thermal conductivity and relatively strong mechanical strength, which suggests that they have great potential in thermal insulation applications. However, because the pore size and density of cellulose aerogels are larger than that of other conventional aerogels such as silica aerogel and resorcinol/formaldehyde (RF) carbon aerogel, and the cellulose matrix also has a larger thermal conductivity, cellulose aerogels have a higher thermal conductivity compared with conventional aerogels. Additionally, the maximum working temperature of cellulose aerogels is less than 300 °C, which limits the development of thermal insulation applications.

### 3.3. Precursor of Carbon Aerogels

Porous carbon aerogels are often used in adsorption, capacitance deionization, catalysis, and supercapacitors due to their large specific surface area, low density, high conductivity, excellent stability, low cost, and long service life [199,200].

The preparation of traditional carbon aerogel involves pyrolyzing the high carbon-content template (resorcinol/formaldehyde aerogel) under high temperature (normally 800~1200 °C), ambient pressure, and an inert atmosphere [201]. The specific surface area of carbon aerogels prepared by resorcinol/formaldehyde (RF) is 706 m^2^/g, while their average pore size is 10.9 nm, and their specific capacitance is 81 F/g [202] in a 1-M H_2_SO_4_ electrolyte solution with a scanning rate of 10 mV/s.

Recently, the use of renewable biomass resources, such as starch, alginate, chitosan, and cellulose as raw materials in the preparation of carbon aerogels has attracted great interest [203,204,205]. Porous cellulose aerogels prepared using cellulose may be a candidate for making carbon aerogels.

Carbon aerogels are obtained through the carbonization of cellulose aerogels in a nitrogen or argon atmosphere by heating the aerogels to 500–1000 °C at a specific rate. The performance of carbon aerogels is related to the performance of cellulose aerogels, the rate of heating, gas atmosphere, carbonization temperature, and post-processing treatment. Table 6 [206,207,208,209,210,211,212,213,214] summarizes the current literature on carbon aerogels derived from cellulose aerogels, and shows the specific surface area, average pore size, specific capacitance, and adsorption performance of carbon aerogels. As shown in Table 6, the specific surface area and average pore size of carbon aerogels derived from cellulose aerogels are 100–1364 m^2^∙g^−1^ and 2–100 nm, respectively, which are values comparable to those of RF–carbon aerogels. Therefore, carbon aerogels derived from cellulose aerogels also have excellent performance in terms of hydrophobicity, flame retardancy, high conductivity, and specific capacity, as well as strong adsorption capacity [90,91,92,203,215,216].

The specific surface area of carbon aerogels obtained by the direct pyrolysis of bamboo fibers is only 26.2 m^2^/g, and their maximum adsorption capacity for oil and organic solvents is only 51 g/g [204]. Therefore, the unique high 3D porosity and large specific surface area of the cellulose aerogel structure are key considerations in the quest to obtain carbon aerogels with good performance. The carbonization of cellulose aerogel causes significant decreases in the volume and mass, because of the removal of O and H atoms [217]. In addition, the activation process can increase the specific surface area of carbon aerogels and improve their pore structure. At present, carbon aerogels derived from cellulose aerogels can be activated in two ways. In one procedure, carbon aerogels are mixed with KOH at a 1:3 mass ratio, followed by 1–3 h of activation after heating the aerogels to 900 °C at a rate of 10 °C/min in a nitrogen atmosphere [149]. In the other procedure, cellulose aerogels are pyrolyzed in a CO_2_ atmosphere, in which the C in the aerogels is oxidized to CO by the presence of CO_2_, while the C=C and oxygen groups on the surface of lignocellulose are eliminated to enhance the surface chemical stability of the final carbon aerogels [154].

Carbon aerogels have an excellent ability to capture CO_2_. Under a carbon dioxide atmosphere, Zhuo et al. have carbonized activated cellulose aerogels to develop a layered and porous carbon aerogel with an excellent CO_2_ absorption capacity (3.42 nmol/g) [206]. In order to improve the absorption capacity of CO_2_, Hu et al. have carbonized cellulose aerogels under a NH_3_ atmosphere and embedded CO_2_-philic nitrogen-containing groups in carbon aerogels to increase their CO_2_ absorption capacity to 4.99 nmol/g [207]. In another method to prepare N-doped carbon aerogels, urea or other nitrogen-containing substances are added into cellulose aerogels, which then undergo pyrolysis in an inert gas to reach a nitrogen content as high as 7.64% [210].

The preparation of cellulose aerogels is a flexible process that provides many convenient opportunities for functionalizing carbon aerogels. For example, the Li group at Northeast Forestry University used cellulose aerogels as precursors to synthesize GO/carbon aerogels and goethite (α-FeOOH)/carbon aerogels with excellent performance in terms of protection against electromagnetic interference. In addition, they have also synthesized NiO/carbon aerogels with excellent electrochemical performance [208,209,211]. The Chen group subjected GO/bacterial cellulose (BC) aerogels to carbonization to develop a carbon aerogel with an adsorption capacity as high as 1002 g/g [212].

Cellulose aerogels provide carbon aerogels with a rich carbon source, a large specific surface area, and mesoporous structures. However, because the porous structure of the physically linked network is fragile during the high temperature pyrolysis process, the modification of cellulose is usually needed [218]. In addition, the solvent exchange and drying processes of cellulose aerogels are time-consuming, while the large-scale production of large cellulose aerogels is difficult, and these factors limit the practical application of cellulose aerogels. Besides, the oxygen in the ring and the OH groups in the cellulose chain make pyrolysis tricky.

### 3.4. Biomedical Materials

Cellulose aerogels are an ultra-light biocompatible material with a 3D network structure associated with high porosity and a high specific surface area. They can be used in drug transport, cell culture, biosensors, and many other biomedical applications.

The 3D cell culture is an important method used in cell biology, regenerative medicine, cell therapy, and drug development. Natural, non-toxic, and biocompatible cellulose aerogels with an interconnected structure of high porosity are ideal scaffolds for 3D cell cultures. Cai et al. have cultured NIH 3T3 cells (mouse embryonic fibroblast cell line) for two weeks on nanocellulose aerogel microspheres cross-linked by Kymene, and found that the number of cells continuously increased, indicating that the cellulose aerogel microspheres could be used as scaffolds for 3D cell culture [84]. Zhang et al. have cultured 3T3 NIH cells on poly(vinyl alcohol)/CNF composite aerogel microspheres and reached the same conclusion [219].

Bacterial adsorption or bacterial growth inhibition can be achieved by fixing antibacterial substances on the surface of cellulose aerogels. Henschen et al. have used a layer-by-layer (LbL) self-assembly technology to adsorb polyvinylamine and polyacrylic acid onto the surface of cellulose aerogels and produced an antibacterial cellulose-based aerogel with contact activity. This material was able to adsorb more than 99.9% of the live bacteria from a bacterial suspension [220]. Uymin et al. have prepared a nanocellulose derivate aerogel with a bacterial inhibition rate of >99.99% by loading lysozymes and silver nanoparticles on the surface of a cationic CNF aerogel [221].

Ethyl cellulose is a water-soluble cellulose derivative that is often used to make vehicles for controlled drug release [222,223]. Choy et al. have used a precision particle fabrication (PPF) technology to produce ethyl cellulose aerogel microspheres under sound and hydrodynamic forces. Such microspheres can provide encapsulation efficiencies of 6.4–51% and 63–80%, respectively, for piroxicam and rhodamine, and near zero-order drug release was observed at 24 h [155]. In addition, pH-controlled or temperature-controlled drug delivery vehicles can be synthesized by modifying cellulose derivate aerogels using cellulose grafting or compounding methods [224,225].

Cellulose aerogels have a connected porous structure and high specific surface areas, so they can be used in biosensors. Edwards et al. used a method based on polypeptide chemistry to graft tripeptide molecules onto a nanocellulose aerogel under the protection of fluorenylmethoxycarbonyl to obtain a polypeptide–nanocellulose aerogel (PepNA). The detection sensitivity of PepNA for human neutrophil elastase is 0.13 U/mL, so it can be used to monitor the level of protease in chronic wounds [226].

Cellular aerogels with biomedical functions can be obtained by surface modification or grafting molecules with specific biological functions onto the molecular chain of cellulose. Due to the organic solvents that are usually used to dissolve cellulose, purifications to avoid toxicity are always needed. Furthermore, the performance stability and reusability of cellular aerogels are currently insufficient for many biomedical applications.

### 3.5. Carrier of Metal Nanoparticles and Metal Oxides

The electronic and chemical properties of metal nanoparticles make them useful for a wide range of applications in electronic devices, optical materials, sensors, and catalysts [227,228,229]. However, the difficulty of immobilizing metal nanoparticles on solid substrates and carrying out separation and processing limits the development and application of metal nanoparticles.

The key problem in the synthesis of metal nanoparticles is to prevent their agglomeration, which can be accomplished by ensuring that cellulose gels have an appropriate nanostructure [120]. In addition, the polar surface of cellulose aerogels is rich in oxygen-containing groups (hydroxyl, carboxyl, and ester groups), which can promote dense nucleation and provide a large number of stable attachment points for metal nanoparticles [230,231]. Therefore, cellulose aerogels with high porosity, a large specific surface area, and good mechanical strength are ideal media for the synthesis and loading of metal nanoparticles.

At present, the metal salt in the nanostructure of cellulose gels is reduced by a hydrothermal method or NaBH_4_ reduction method. In this way, cellulose aerogels loaded with metal nanoparticles can be obtained after drying. The diameter of these metal nanoparticles is typically <100 nm, and their number and size can be determined by controlling the metal salt concentration, temperature, and reaction time.

Metal nanoparticles can provide cellulose aerogels with excellent performance. In addition, the unique 3D network structure of aerogels can also strengthen the catalytic and conductive capabilities of metal particles. Keshipour and Khezerloo deposited gold nanoparticles onto the surface of regenerated aerogels to obtain a catalyst that efficiently catalyzed styrene epoxidation (96%, 1 h) [232]. Then, they prepared a new cellulose derivative aerogel by modifying cellulose aerogel with chloroacetic acid, dimercaprol, and Au(ш) to get an efficient heterogeneous catalyst in the oxidation reactions of aliphatic, benzyl alcohol, and ethylbenzene [233]. Thiruvengadam and Vitta prepared a Ni–BC aerogel nanocomposite with thermally sensitive magnetic behavior [234]. The Yao group developed an aerogel consisting of pressure-sensitive conductive material Ag/CNF by combining a silver mirror reaction with ultrasonic treatment [235]. The Zhang group prepared a polyaniline (PANI)/Ag/CNF elastic supercapacitor (176 mF/cm^2^ at 10 mV·s^−1^) by electroplating a layer of PANI on the surface of an Ag/CNF aerogel [236].

Unlike metal nanoparticles, metal oxides are typically deposited on the surface of aerogels by chemical vapor deposition or in situ precipitation. For example, the Kettunen and Korhonen groups coated a uniform thin layer of 7-nm titanium dioxide (TiO_2_) on the surface of aerogels using a chemical vapor deposition method and an atomic layer deposition method, respectively, and obtained TiO_2_-cellulose aerogels with strong light-controlled water absorption capacity or strong oil adsorption capacity [237,238].

In addition, metal (metal oxide) aerogels may also be prepared by immersing nanoparticle–cellulose aerogels in cellulose solvents or by pyrolyzing the aerogels in oxygen to remove the cellulose [239,240]. Although the performance of metal (metal oxide) aerogels obtained this way is better than those obtained through other means, the time required to produce such aerogels is several fold greater.

### 3.6. Other Applications

The low ionic conductivity of PP separators has greatly limited their application in lithium ion batteries. Liao et al. used ice-separation induced self-assembly to coat a hydroxyethyl cellulose aerogel on a commercial PP separator and greatly improved the size stability of the PP separator, as well as the adsorption and retention rate of electrolytes, thus increasing the ionic conductivity and reusability of the PP separator [241].

In actual applications, phase-change composite materials must have high thermal conductivity, a high latent heat of melting, and good structural stability. Yang et al. impregnated polyethylene glycol (PEG) onto the surface of a cellulose/graphite nanoplatelet (GNP) aerogel to prepare a phase-change composite material with high thermal conductivity (1.35 W∙m^−1^∙K^−1^) and high latent heat of melting (156.1 J∙g^−1^) [242]. This type of surface impregnation method is often used in the functionalization of cellulose aerogels. Pääkkö et al. impregnated a conductive polymer, polyaniline-sodium dodecylbenzenesulfonate, on the surface of a natural cellulose aerogel to confer high conductivity (approximately 1 × 10^−2^ S∙cm^−1^) [80].

In addition, the nanoscale and connected nature of the porous networks in cellulose aerogels, as well as high specific surface areas, confer an excellent filtration performance and provide a large number of contactable sites, making them ideal materials for producing air filters and sensors [58,243,244].

## 4. Conclusions and Prospect

Cellulose aerogels have the environmentally-friendly renewability, biocompatibility, and biodegradability of cellulose, but also have excellent properties such as low density, high porosity, and a high specific surface area. Cellulose aerogels are particularly well suited for applications in the areas of adsorption and separation of biomedical and thermal insulation materials, as well as many other fields.

However, there are still some issues regarding the preparation and modification of cellulose aerogels. (1) First, the cost of nanocellulose and bacterial cellulose is high, and nanocellulose is prone to self-agglomeration during the drying process. In addition, it is difficult to recover cellulose solvents during the preparation of regenerated cellulose aerogels, and the solvent exchange process tends to be very time-consuming; (2) Second, some modification methods for cellulose aerogels, such as modification by a silane coupling agent, are complex and have a relatively high cost; (3) The structural strength and stability performance (such as thermal stability and capability for repeated adsorption) of cellulose aerogels still cannot meet the requirements of many actual applications.

Therefore, the following problems should be addressed by the future research and development of cellulose aerogels. Efficient, inexpensive, environmentally-friendly and non-toxic cellulose solvent systems are necessary to improve the dissolution efficiency of cellulose. In addition, the sol–gel and solvent exchange processes must be expedited to shorten the production cycle. Low-cost equipment, as well as easy and safe gel drying methods, should be explored. Finally, the potential for the performance and stability of cellulose aerogels to be improved by physical mixing or chemical modifications should be assessed.

## Figures and Tables

**Table 1:** Properties of natural cellulose aerogels.

Materials	Drying Method	Density (g∙cm^−3^)	Porosity (%)	Specific Surface Area (m^2^∙g^−1^)	Compression Modulus (kPa)	Ref.
Bleached cellulose fibers, CNC, TEMPO-NCF	Freeze dried	-	-	143–162	13–176	[62]
Cellulose whisker, clay, PVA	Freeze dried	0.01–0.101	-	-	18–788	[77]
CNC	Freeze dried	-	-	91.47–93.89	-	[78]
BC	ScCO_2_ dried	0.008	-	200	-	[79]
CNC	Freeze dried	0.02–0.03	95–98.7	20–66	200–240	[80]
NCF	Freeze dried	0.003	-	20.09	37	[81]
CNC	ScCO_2_ dried	-	-	260–353	-	[72]
CNC	ScCO_2_ dried	0.078–0.155	91–95	216–605	-	[82]
NCF	Freeze dried	0.0005–0.01	99.38–99.97	-	0.2–5.2	[83]
NCF, Kymene	Freeze dried	0.0018–0.005	-	389	-	[84]
BC, GO	Freeze dried	-	99.84–99.92	-	-	[85]
BC, silica	Freeze dried	0.007–0.229	89–99.6	129–541.1	270–16670	[86]
NCF	Freeze dried	0.02	98.6	-	-	[87]
NCF	Freeze dried	0.0053–0.03	98.2–99.7	11–15	-	[88]
NCF, SiO_2_	Freeze dried	0.055–0.295	85.15–96.46	11.3–700.1	1740–5930	[89]
BC	Freeze dried	0.009–0.01	-	-	-	[90]
NCF	Freeze dried	0.025	97.8	-	-	[91]
NCF	Freeze dried	0.02	-	-	-	[92]
CNC, SiO_2_	Ambient pressure drying	0.137–0.151	-	620–688	-	[93]

CNC, cellulose nanocrystals; TEMPO-NCF, 2,2,6,6-tetramethylpiperidine-1-oxyl (TEMPO)-mediated nanofibrillated cellulose (NCF); PVA, poly(vinyl alcohol); CNC, cellulose nanocrystals; BC, bacterial cellulose; GO, graphene oxide.

**Table 2:** Properties of regenerated cellulose aerogels.

Materials	Solvent	Drying Method	Density (g∙cm^−3^)	Porosity (%)	Specific Surface Area (m^2^∙g^−1^)	Compression Modulus (kPa)	Ref.
Wood pulp	ILs	scCO_2_ dried	0.058	94–96	315	-	[4]
Cellulose powder/GO	NaOH/thiourea	Freeze dried	-	-	-	870–1130	[73]
MCC/lignin	8% NaOH	scCO_2_ dried	0.1–0.135	-	200	-	[74]
MCC	8% NaOH	scCO_2_ dried	0.06–0.3	91–96	200–300	-	[67]
Cotton linter	NMMO Ca(SCN)_2_/LiCl TBAF/DMSO [EMIm][OAc]/DMSO	scCO_2_ dried	0.03–0.067	95.5–98.1	190–328	22–240	[105]
Wood pulp	NMMO	scCO_2_ dried	0.014–0.5	-	50–420	-	[106]
Cellulose powder	Ca(SCN)_2_	scCO_2_ dried	0.009–0.137	91–99	120–230	1400–16200	[107]
Cellulose powder	Ca(SCN)_2_	Freeze dried	-	-	160–190	-	[108]
Cotton linter	NaOH/thiourea	Freeze dried	0.2–0.4	<84.88	-	5700–8200	[109]
Paper pulp	Alkali/urea	scCO_2_ dried	0.03–0.14	89.7–97	291–485	-	[110]
MCC	ZnCl_2_	scCO_2_ dried	0.082–0.245		212–864	800	[111]
Wood	ILs	scCO_2_ dried	0.06–0.2	-	150–200	1000–10000	[112]
MCC	LiCl/DMAc	Freeze dried	0.12–0.35	-	-	-	[113]
Bagasse	LiCl/DMSO	Freeze dried	0.088–0.236	84.4–94.2	119–185	-	[114]
MCC	LiCl/DMSO	Freeze dried	0.068–0.137	-	185–213	-	[115]
Cellulose fibers	Ca(SCN)_2_	Freeze dried or scCO_2_ dried	0.01–0.06	-	80–250	2000	[116]
Wood	ILs	scCO_2_ dried	0.141–0.157	97	-	-	[117]
Wood	ILs	scCO_2_ dried	0.095–0.143	-	2–80.7	-	[118]
Cellulose	NMMO	scCO_2_ dried	0.05–0.26	-	172–284	-	[119]
Paper pulp	LiOH/urea	scCO_2_ dried	0.12–0.17	95	363–406	-	[120]
MCC	ILs	Freeze dried	-	-	-	-	[121]
Cotton linter	NaOH/urea	Freeze dried	0.027–0.056	96.3–98.2	-	-	[122]
Waste newspaper	ILs	Freeze dried	0.017–0.029	98.2–98.9	296–412	-	[123]
Recycled cellulose	NaOH/urea	Freeze dried	0.04	94.8	-	11	[124]
Waste newspaper	ILs	Freeze dried	0.02–0.029	96.8		-	[125]
Recycled cellulose	NaOH/urea	Freeze dried	0.04	97.3	-	-	[126]
Cotton linter	NaOH/urea	Freeze dried	0.0196	98.7	-	-	[127]
Wood pulp	ILs	Freeze dried	<0.034	>98.5	-	-	[128]
Wheat straw	NaOH/PEG	Freeze dried	0.04	-	36.46–101.13	-	[129]
Plant	NaOH/PEG	Freeze dried	0.053–0.092	-	63–152.5	-	[130]
Cellulose/SiO_2_	Alkali/urea	scCO_2_ dried	0.14–0.58	70–92	356–652	7900–12000	[131]
Cellulose/SiO_2_	ILs/DMSO	scCO_2_ dried	0.125–0.225	87–94	290–975	-	[132]
Cellulose/SiO_2_	Ca(SCN)_2_	scCO_2_ dried	0.041–0.163	-	-	1500–4200	[133]
Cellulose: lignin, xylan	ILs	scCO_2_ dried	0.025–0.114	-	108–539	-	[134]

GO, graphene oxide; MCC, microcrystalline cellulose; ILs, ionic liquids; NMMO, *N*-methylmorpholine-*N*-oxide; TBAF, tetrabutylammonium fluoride; DMSO, dimethyl sulfoxide; [EMIm][OAc], 1-Ethyl-3-methyl-1Himidazolium acetate; DMAc, dimethylacetamide; PEG, polyethylene glycol.

**Table 3:** Properties of cellulose derivative aerogels.

Materials	Solvent	Drying Method	Density (g∙cm^−3^)	Porosity (%)	Specific Surface Area (m^2^∙g^−1^)	Compression Modulus (kPa)	Ref.
Cellulose derivative aerogels							
TAC	Dioxane/Isopropanol	scCO_2_ dried	0.005–0.05	96.1–99.6	229–958	-	[147]
CA	Acetone	scCO_2_ dried	0.25–0.85	-	140–250	-	[148]
CMC	Water	Freeze dried	-	-	-	-	[149]
CMC	Water	Freeze dried	0.062–0.12	-	-	830–3442	[150]
HPMC	Water	Freeze dried	0.018–0.023	-	-	111–133	[151]
CA	Acetone	scCO_2_ dried	0.16	-	-	-	[154]
EC	Dichloromethane	Freeze dried	-	-	-	-	[155]
CMC/CNF	Water	Freeze dried	0.05–0.109	93.19–96.84	-	1000–8700	[156]
Nanocellulose derivative aerogels							
CNF-MA	-	Freeze dried	0.0112–0.0315	-	19.5	120–411	[152]
BMCC/CMCT	-	Freeze dried	-	98.8	-	-	[153]
TEMPO-CNF	-	Freeze dried	0.008–0.187	98.8–99.5	12.72–117.8	-	[157]
TEMPO-CNF	-	Freeze dried	0.014–0.105	92.8–99	153–284	34.9–2800	[158]
TEMPO-CNF	-	Freeze dried	-	-	94–319	-	[159]
TEMPO-CNF	-	Freeze dried	0.0069–0.0083		123–209	94–209	[160]
TEMPO-CNF/PVA	-	Freeze dried	0.0047–0.0165	98.7–99.7	35.1–117	-	[161]
TEMPO-CNF	-	Freeze dried	0.0017–0.0081	99.5–99.9	10.9	54.5–25.3	[162]
Hydrophobic CNF	-	Freeze dried	0.0232	98.5	18.4	-	[163]
TEMPO-CNF/Eumelanin	-	Freeze dried	0.04	97.5	-	-	[164]

TAC, triacetyl cellulose; CA, cellulose acetate; CMC, carboxymethylcellulose; HPMC, hydroxypropyl methylcellulose; EC, ethyl cellulose; CNF, cellulose nanofibers; CNF-MA, maleic acid-grafted CNF; BMCC, bifunctional (aldehyde and carboxyl) nanocellulose; CMCT, cross-linked carboxymethyl chitosan; TEMPO, 2,2,6,6-tetramethylpiperidine-1-oxyl; PVA, poly(vinyl alcohol).

**Table 4:** Hydrophobic treatments and absorption capacity of cellulose aerogels. GO: graphene oxide.

Classification	Hydrophobic Treatment	WCA (°)	Porosity (%)	Specific Surface Area (m^2^/g)	Absorption Capacity (g/g)	Ref.
Natural cellulose aerogel	CVD of TMCS	135	-	20.09	52	[81]
GO/natural cellulose aerogel	-	-	99.86, 99.84	-	135–150	[85]
Regenerated cellulose aerogel	CVD of MTCS	141	98.0	-.	40.16–59.32	[122]
Regenerated cellulose aerogel	CVD of TMCS	135	98.2	405	26–45	[123]
Regenerated cellulose aerogel	Water repellent spry or CVD of MTMS	130.7, 135.2	94.8	-	18–20	[124]
Regenerated cellulose aerogel	CVD of MTCS	136	96.8	-	12–22	[125]
Regenerated cellulose aerogel	CVD of MTMS	145	97.3	-	18.4–20.5	[126]
Regenerated cellulose aerogel	Cold plasma technology	150	98.7	-	34.5	[127]
Regenerated cellulose aerogel	Plasma treatment and subsequent silane modification	>156	>98.5	-	14–42	[128]
Regenerated cellulose aerogel	CVD of TMCS	138	-	36.46–101	16.8–18.7	[129]
Regenerated cellulose aerogel	CVD of MTCS	-	-	63.3–152.5	13.5–20.6	[130]
Cellulose derivate aerogel	Cross-linking with diisocyanate	-	-	216–228	42.4–54.47	[160]
Cellulose derivate aerogel microsphere	CVD of MTCS	-	98.7–99.7	35.1–117	54–140	[161]
Cellulose derivate aerogel	Vapor deposition with triethoxyl(octyl) silane	-	99.5–99.9	10.9	139–375	[162]
Cellulose derivate aerogel	Polymerization with a monomer dropwise-feeding method	-	98.5	18.4	29.9–46.6	[163]
Natural cellulose aerogel	CVD of MTMS	150.8–153.5	97.2–99.4	-	40.4–95	[183]
Natural cellulose aerogel	CVD of MTMS	142.8	99.43–99.66	-	40–100	[184]
GO/natural cellulose aerogel	CVD of DDTS	150.3	-	47.3	80–197	[185]

CVD, chemical vapor deposition; TMCS, trimethylchlorosilane; MTMS, methyltrimethoxysilane; MTCS, methyltrichlorosilane; DDTS, *n*-dodecyltriethoxysilane.

**Table 5:** Thermal conductivity of cellulose aerogels.

Classification	Density (g∙cm^−3^)	Pore Size (nm)	Technique	Conductivity (W∙m^−1^∙K^−1^)	Ref.
Natural cellulose aerogel	-	5–13	Hot filament	0.023–0.028	[62]
Natural cellulose aerogel, SiO_2_	0.007–0.229	-	Transient plate method	0.0295–0.0369	[86]
Natural cellulose aerogel, SiO_2_	0.055–0.295	-	Double plate method	0.0226	[87]
Regenerated cellulose aerogel	0.009–0.137	10–100	Transient plane source	0.04–0.075	[107]
Regenerated cellulose aerogel	0.2–0.4	-	Conductometer	0.029–0.046	[109]
Regenerated cellulose aerogel	0.095–0.143	10.5–28.9	Model digital thermal diffusivity instrument	0.03–0.137	[118]
Regenerated cellulose aerogel	0.04	-	Transient plate method	0.029, 0.032	[124]
Regenerated cellulose aerogel, SiO_2_	0.14–0.58	3–20	-	0.025–0.045	[131]
Regenerated cellulose aerogel, SiO_2_	0.125–0.225	-	Steady state method	0.026–0.033	[132]
Regenerated cellulose aerogel, SiO_2_	0.041–0.103	-	HotDisk™	0.04–0.052	[133]
Cellulose derivate aerogel	0.25–0.85	13–25	Hot-wire method	0.029	[148]
Cellulose derivate aerogel	0.05–0.109	5000–40,000	Transient plane source method	0.040–0.0532	[156]
Cellulose derivate aerogel	0.012–0.033	10–100	Hot strip	0.018–0.028	[169]
Natural cellulose aerogel, SiO_2_	0.007–0.201	-	Transient plate method	0.029–0.037	[192]

**Table 6:** Properties of carbon aerogels derived from cellulose aerogels.

Cellulose Aerogel Precursor	Specific Surface Area (m^2^∙g^−1^)	Mean Pore Diameter (nm)	Specific Capacitance (F/g)	Adsorption Capacity	Ref.
Natural cellulose aerogel	-	10–20	-	Organic solvents and oils: 106 to 312 times its own weight	[90]
Natural cellulose aerogel	145–521	10–20		Oil: 55.8–86.6 g/g	[91]
Natural cellulose aerogel	-	-	-	Oil: 67.26–94.18	[92]
Cellulose derivate aerogel	230–428	2.81–4.25	92.34–152.6	Methylene blue: 249.6 mg/g Malachite green: 245.3 mg/g	[149]
Cellulose derivate aerogel	400–450	-	-	-	[154]
Regenerated cellulose aerogel	113	7.2	-	-	[203]
Regenerated cellulose aerogel	859–1364	-	328	CO_2_: 3.01–3.42 mmol/g	[206]
Regenerated cellulose aerogel	496–615	2–3	225	CO_2_: 4.99 mmol/g	[207]
GO/Regenerated cellulose aerogel	-	-	-	-	[208]
Regenerated cellulose aerogel	-	-	-	-	[209]
Regenerated cellulose aerogel	450–853	20–100	129–193	-	[210]
Regenerated cellulose aerogel	170.05	3.44–4.52	73.18–294.01	-	[211]
GO/Natural cellulose aerogel	110.4	-	-	Organic solvents and oils: 393–1002 g/g	[212]
Cellulose derivate aerogel	742.34	2–75	-	-	[213]
Cellulose derivate aerogel	185	2–259	-	Organic solvents and oil: 20 g/g	[214]

## References

[1] Sticklen M.B. (2008). Plant genetic engineering for biofuel production: Towards affordable cellulosic ethanol. Nat. Rev. Genet..

[2] Klemm D., Heublein B., Fink H.P., Bohn A. (2005). Cellulose: Fascinating biopolymer and sustainable raw material. Angew. Chem. Int. Ed..

[3] Habibi Y., Lucia L.A., Rojas O.J. (2010). Cellulose nanocrystals: Chemistry, self-assembly, and applications. Chem. Rev..

[4] Tsioptsias C., Stefopoulos A., Kokkinomalis I., Papadopoulou L., Panayiotou C. (2008). Development of micro- and nano-porous composite materials by processing cellulose with ionic liquids and supercritical CO_2_. Green Chem..

[5] Wang S., Lu A., Zhang L. (2016). Recent advances in regenerated cellulose materials. Prog. Polym. Sci..

[6] Kistler S.S. (1931). Coherent expanded aerogels and jellies. Nature.

[7] Kistler S.S. (1932). Coherent Expanded Aerogels. Rubber Chem. Technol..

[8] Leventis N., Sotiriou-Leventis C., Zhang G., Rawashdeh A.M.M. (2002). Nanoengineering Strong Silica Aerogels. Nano Lett..

[9] Masson O., Rieux V., Guinebretière R., Dauger A. (1996). Size and shape characterization of TiO_2_aerogel nanocrystals. Nanostruct. Mater..

[10] Baumann T.F., Kucheyev S.O., Gash A.E., Satcher J.H. (2005). Facile synthesis of a crystalline, high-surface-area SnO_2_ aerogel. Adv. Mater..

[11] Le D.B. (1996). High Surface Area V_2_O_5_ Aerogel Intercalation Electrodes. J. Electrochem. Soc..

[12] Corrias A., Casula M.F., Falqui A., Paschina G. (2004). Preparation and Characterization of FeCo-Al_2_O_3_ and Al_2_O_3_ Aerogels. J. Sol-Gel Sci. Technol..

[13] Al-Muhtaseb S.A., Ritter J.A. (2003). Preparation and properties of resorcinol-formaldehyde organic and carbon gels. Adv. Mater..

[14] Yamashita J., Ojima T., Shioya M., Hatori H., Yamada Y. (2003). Organic and carbon aerogels derived from poly(vinyl chloride). Carbon N. Y..

[15] Daniel C., Sannino D., Guerra G. (2008). Syndiotactic polystyrene aerogels: Adsorption in amorphous pores and absorption in crystalline nanocavities. Chem. Mater..

[16] Guo H., Meador M.A.B., McCorkle L., Quade D.J., Guo J., Hamilton B., Cakmak M. (2012). Tailoring properties of cross-linked polyimide aerogels for better moisture resistance, flexibility, and strength. ACS Appl. Mater. Interfaces.

[17] García-González C.A., Alnaief M., Smirnova I. (2011). Polysaccharide-based aerogels—Promising biodegradable carriers for drug delivery systems. Carbohydr. Polym..

[18] Deze E.G., Papageorgiou S.K., Favvas E.P., Katsaros F.K. (2012). Porous alginate aerogel beads for effective and rapid heavy metal sorption from aqueous solutions: Effect of porosity in Cu^2+^ and Cd^2+^ ion sorption. Chem. Eng. J..

[19] Betz M., García-González C.A., Subrahmanyam R.P., Smirnova I., Kulozik U. (2012). Preparation of novel whey protein-based aerogels as drug carriers for life science applications. J. Supercrit. Fluids.

[20] Chang X., Chen D., Jiap X. (2008). Chitosan-based aerogels with high adsorption performance. J. Phys. Chem. B.

[21] Salam A., Venditti R.A., Pawlak J.J., El-Tahlawy K. (2011). Crosslinked hemicellulose citrate-chitosan aerogel foams. Carbohydr. Polym..

[22] Fairén-Jiménez D., Carrasco-Marín F., Moreno-Castilla C. (2006). Porosity and surface area of monolithic carbon aerogels prepared using alkaline carbonates and organic acids as polymerization catalysts. Carbon N. Y..

[23] Aliev A.E., Oh J., Kozlov M.E., Kuznetsov A.A., Fang S., Fonseca A.F., Ovalle R., Lima M.D., Haque M.H., Gartstein Y.N. (2009). Giant-Stroke, Superelastic Carbon Nanotube Aerogel Muscles. Science.

[24] Worsley M.A., Pauzauskie P.J., Olson T.Y., Biener J., Satcher J.H., Baumann T.F. (2010). Synthesis of graphene aerogel with high electrical conductivity. J. Am. Chem. Soc..

[25] Maleki H. (2016). Recent advances in aerogels for environmental remediation applications: A review. Chem. Eng. J..

[26] Hrubesh L.W. (1998). Aerogel applications. J. Non-Cryst. Solids.

[27] Bheekhun N., Abu Talib A.R., Hassan M.R. (2013). Aerogels in aerospace: An overview. Adv. Mater. Sci. Eng..

[28] Vareda J.P., Valente A.J.M., Durães L. (2016). Heavy metals in Iberian soils: Removal by current adsorbents/amendments and prospective for aerogels. Adv. Colloid Interface Sci..

[29] Stergar J., Maver U. (2016). Review of aerogel-based materials in biomedical applications. J. Sol-Gel Sci. Technol..

[30] Maleki H., Durães L. (2014). An overview on silica aerogels synthesis and different mechanical reinforcing strategies. J. Non-Cryst. Solids.

[31] Katti A., Shimpi N., Roy S., Lu H., Fabrizio E.F., Dass A., Capadona L.A., Leventis N. (2006). Chemical, physical, and mechanical characterization of isocyanate cross-Linked amine-modified silica aerogels. Chem. Mater..

[32] Nechyporchuk O., Belgacem M.N., Bras J. (2015). Production of cellulose nanofibrils: A review of recent advances. Ind. Crops Prod..

[33] Moon R.J., Martini A., Nairn J., Simonsen J., Youngblood J. (2011). Cellulose nanomaterials review: Structure, properties and nanocomposites. Chem. Soc. Rev..

[34] Aegerter M., Leventis N., Koebel M. (2011). Aerogels Handbook (Advances in Sol-Gel Derived Materials and Technologies).

[35] Zhao S., Malfait W.J., Guerrero Alburquerque N., Koebel M.M., Nyström G. (2018). Biopolymer Aerogels: Chemistry, Properties and Applications. Angew. Chem. Int. Ed..

[36] Liu H., Geng B., Chen Y., Wang H. (2017). Review on the Aerogel-Type Oil Sorbents Derived from Nanocellulose. ACS Sustain. Chem. Eng..

[37] De France K.J., Hoare T., Cranston E.D. (2017). Review of Hydrogels and Aerogels Containing Nanocellulose. Chem. Mater..

[38] Fernandes E.M., Pires R.A., Mano J.F., Reis R.L. (2013). Bionanocomposites from lignocellulosic resources: Properties, applications and future trends for their use in the biomedical field. Prog. Polym. Sci..

[39] Eichhorn S.J., Dufresne A., Aranguren M., Marcovich N.E., Capadona J.R., Rowan S.J., Weder C., Thielemans W., Roman M., Renneckar S. (2010). Review: Current international research into cellulose nanofibres and nanocomposites. J. Mater. Sci..

[40] Jianan C., Shaoqiong Y., Jinyue R. (1996). A study on the preparation, structure, and properties of microcrystalline cellulose. J. Macromol. Sci. Part A Pure Appl. Chem..

[41] Virtanen T., Svedström K., Andersson S., Tervala L., Torkkeli M., Knaapila M., Kotelnikova N., Maunu S.L., Serimaa R. (2012). A physico-chemical characterisation of new raw materials for microcrystalline cellulose manufacturing. Cellulose.

[42] Reddy N., Yang Y. (2009). Properties and potential applications of natural cellulose fibers from the bark of cotton stalks. Bioresour. Technol..

[43] Wang X., Li H., Cao Y., Tang Q. (2011). Cellulose extraction from wood chip in an ionic liquid 1-allyl-3-methylimidazolium chloride (AmimCl). Bioresour. Technol..

[44] Cara C., Ruiz E., Ballesteros I., Negro M.J., Castro E. (2006). Enhanced enzymatic hydrolysis of olive tree wood by steam explosion and alkaline peroxide delignification. Process Biochem..

[45] Abe K., Yano H. (2009). Comparison of the characteristics of cellulose microfibril aggregates of wood, rice straw and potato tuber. Cellulose.

[46] Sun J.X., Sun X.F., Zhao H., Sun R.C. (2004). Isolation and characterization of cellulose from sugarcane bagasse. Polym. Degrad. Stab..

[47] Trache D., Hussin M.H., Hui Chuin C.T., Sabar S., Fazita M.R.N., Taiwo O.F.A., Hassan T.M., Haafiz M.K.M. (2016). Microcrystalline cellulose: Isolation, characterization and bio-composites application—A review. Int. J. Biol. Macromol..

[48] Siqueira G., Bras J., Dufresne A. (2010). Cellulosic bionanocomposites: A review of preparation, properties and applications. Polymers.

[49] Lee S., Jeong M.-J., Kang K.-Y. (2015). Preparation of cellulose aerogels as a nano-biomaterial from lignocellulosic biomass. J. Korean Phys. Soc..

[50] Jeong M.-J., Lee S., Kang K.-Y., Potthast A. (2015). Changes in the structure of cellulose aerogels with depolymerization. J. Korean Phys. Soc..

[51] De Oliveira Barud H.G., da Silva R.R., da Silva Barud H., Tercjak A., Gutierrez J., Lustri W.R., de Oliveira O.B., Ribeiro S.J.L. (2016). A multipurpose natural and renewable polymer in medical applications: Bacterial cellulose. Carbohydr. Polym..

[52] Ullah H., Santos H.A., Khan T. (2016). Applications of bacterial cellulose in food, cosmetics and drug delivery. Cellulose.

[53] Foresti M.L., Vázquez A., Boury B. (2017). Applications of bacterial cellulose as precursor of carbon and composites with metal oxide, metal sulfide and metal nanoparticles: A review of recent advances. Carbohydr. Polym..

[54] Kobayashi S., Sakamoto J., Kimura S. (2001). In vitro synthesis of cellulose and related polysaccharides. Prog. Polym. Sci..

[55] Habibi Y. (2014). Key advances in the chemical modification of nanocelluloses. Chem. Soc. Rev..

[56] Roy D., Semsarilar M., Guthrie J.T., Perrier S. (2009). Cellulose modification by polymer grafting: A review. Chem. Soc. Rev..

[57] Abdul Khalil H.P.S., Bhat A.H., Yusra A.F.I. (2012). Green composites from sustainable cellulose nanofibrils: A review. Carbohydr. Polym..

[58] Brinchi L., Cotana F., Fortunati E., Kenny J.M. (2013). Production of nanocrystalline cellulose from lignocellulosic biomass: Technology and applications. Carbohydr. Polym..

[59] Kim J.H., Shim B.S., Kim H.S., Lee Y.J., Min S.K., Jang D., Abas Z., Kim J. (2015). Review of nanocellulose for sustainable future materials. Int. J. Precis. Eng. Manuf. Green Technol..

[60] Klemm D., Kramer F., Moritz S., Lindström T., Ankerfors M., Gray D., Dorris A. (2011). Nanocelluloses: A new family of nature-based materials. Angew. Chem. Int. Ed..

[61] Ahmadi M., Madadlou A., Saboury A.A. (2016). Whey protein aerogel as blended with cellulose crystalline particles or loaded with fish oil. Food Chem..

[62] Seantier B., Bendahou D., Bendahou A., Grohens Y., Kaddami H. (2016). Multi-scale cellulose based new bio-aerogel composites with thermal super-insulating and tunable mechanical properties. Carbohydr. Polym..

[63] Nguyen B.N., Cudjoe E., Douglas A., Scheiman D., McCorkle L., Meador M.A.B., Rowan S.J. (2016). Polyimide Cellulose Nanocrystal Composite Aerogels. Macromolecules.

[64] Hüsing N., Schubert U. (1998). Aerogels—Airy Materials: Chemistry, Structure, and Properties. Angew. Chem. Int. Ed..

[65] Gesser H.D., Goswami P.C. (1989). Aerogels and Related Porous Materials. Chem. Rev..

[66] Gurav J.L., Jung I., Park H., Kang E.S., Nadargi D.Y. (2010). Silica Aerogel: Synthesis and Applications. J. Nanomater..

[67] Gavillon R., Budtova T. (2008). Aerocellulose: New highly porous cellulose prepared from cellulose-NaOH aqueous solutions. Biomacromolecules.

[68] Biganska O., Navard P. (2009). Morphology of cellulose objects regenerated from cellulose-*N*-methylmorpholine *N*-oxide-water solutions. Cellulose.

[69] Shen X., Shamshina J.L., Berton P., Gurau G., Rogers R.D. (2016). Hydrogels based on cellulose and chitin: Fabrication, properties, and applications. Green Chem..

[70] Sannino A., Demitri C., Madaghiele M. (2009). Biodegradable cellulose-based hydrogels: Design and applications. Materials.

[71] Wang X., Zhang Y., Jiang H., Song Y., Zhou Z., Zhao H. (2016). Fabrication and characterization of nano-cellulose aerogels via supercritical CO_2_ drying technology. Mater. Lett..

[72] Wang X., Zhang Y., Jiang H., Song Y., Zhou Z., Zhao H. (2017). Tert-butyl alcohol used to fabricate nano-cellulose aerogels via freeze-drying technology. Mater. Res. Express.

[73] Zhang J., Cao Y., Feng J., Wu P. (2012). Graphene Oxide Sheet Induced Gelation of Cellulose and Promoted Mechanical Properties of Composite Aerogels, SI. J. Phys. Chem. C.

[74] Sescousse R., Smacchia A., Budtova T. (2010). Influence of lignin on cellulose-NaOH-water mixtures properties and on Aerocellulose morphology. Cellulose.

[75] Zugenmaier P. (2001). Comformation and packing of various crystalline cellulose fibers. Prog. Polym. Sci..

[76] OSullivan A.C. (1997). Cellulose: The structure slowly unravels. Cellulose.

[77] Gawryla M.D., van den Berg O., Weder C., Schiraldi D.A. (2009). Clay aerogel/cellulose whisker nanocomposites: A nanoscale wattle and daub. J. Mater. Chem..

[78] Rahbar Shamskar K., Heidari H., Rashidi A. (2016). Preparation and evaluation of nanocrystalline cellulose aerogels from raw cotton and cotton stalk. Ind. Crops Prod..

[79] Liebner F., Haimer E., Wendland M., Neouze M.A., Schlufter K., Miethe P., Heinze T., Potthast A., Rosenau T. (2010). Aerogels from unaltered bacterial cellulose: Application of scCO_2_ drying for the preparation of shaped, ultra-lightweight cellulosic aerogels. Macromol. Biosci..

[80] Pääkkö M., Vapaavuori J., Silvennoinen R., Kosonen H., Ankerfors M., Lindström T., Berglund L.A., Ikkala O. (2008). Long and entangled native cellulose I nanofibers allow flexible aerogels and hierarchically porous templates for functionalities. Soft Matter.

[81] Xiao S., Gao R., Lu Y., Li J., Sun Q. (2015). Fabrication and characterization of nanofibrillated cellulose and its aerogels from natural pine needles. Carbohydr. Polym..

[82] Heath L., Thielemans W. (2010). Cellulose nanowhisker aerogels. Green Chem..

[83] Zhang X., Yu Y., Jiang Z., Wang H. (2015). The effect of freezing speed and hydrogel concentration on the microstructure and compressive performance of bamboo-based cellulose aerogel. J. Wood Sci..

[84] Cai H., Sharma S., Liu W., Mu W., Liu W., Zhang X., Deng Y. (2014). Aerogel microspheres from natural cellulose nanofibrils and their application as cell culture scaffold. Biomacromolecules.

[85] Wang Y., Yadav S., Heinlein T., Konjik V., Breitzke H., Buntkowsky G., Schneider J.J., Zhang K. (2014). Ultra-light nanocomposite aerogels of bacterial cellulose and reduced graphene oxide for specific absorption and separation of organic liquids. RSC Adv..

[86] Sai H., Xing L., Xiang J., Cui L., Jiao J., Zhao C., Li Z., Li F., Zhang T. (2014). Flexible aerogels with interpenetrating network structure of bacterial cellulose-silica composite from sodium silicate precursor via freeze drying process. RSC Adv..

[87] Jin H., Kettunen M., Laiho A., Pynnönen H., Paltakari J., Marmur A., Ikkala O., Ras R.H.A. (2011). Superhydrophobic and superoleophobic nanocellulose aerogel membranes as bioinspired cargo carriers on water and oil. Langmuir.

[88] Aulin C., Netrval J., Wågberg L., Lindström T. (2010). Aerogels from nanofibrillated cellulose with tunable oleophobicity. Soft Matter.

[89] Fu J., Wang S., He C., Lu Z., Huang J., Chen Z. (2016). Facilitated fabrication of high strength silica aerogels using cellulose nanofibrils as scaffold. Carbohydr. Polym..

[90] Wu Z.Y., Li C., Liang H.W., Chen J.F., Yu S.H. (2013). Ultralight, flexible, and fire-resistant carbon nanofiber aerogels from bacterial cellulose. Angew. Chem. Int. Ed..

[91] Meng Y., Young T.M., Liu P., Contescu C.I., Huang B., Wang S. (2015). Ultralight carbon aerogel from nanocellulose as a highly selective oil absorption material. Cellulose.

[92] Meng Y., Wang X., Wu Z., Wang S., Young T.M. (2015). Optimization of cellulose nanofibrils carbon aerogel fabrication using response surface methodology. Eur. Polym. J..

[93] Li M., Jiang H., Xu D., Yang Y. (2017). A facile method to prepare cellulose whiskers–silica aerogel composites. J. Sol-Gel Sci. Technol..

[94] Mondal S. (2017). Preparation, properties and applications of nanocellulosic materials. Carbohydr. Polym..

[95] Abdul Khalil H.P.S., Davoudpour Y., Islam M.N., Mustapha A., Sudesh K., Dungani R., Jawaid M. (2014). Production and modification of nanofibrillated cellulose using various mechanical processes: A review. Carbohydr. Polym..

[96] Iwamoto S., Nakagaito A.N., Yano H., Nogi M. (2005). Optically transparent composites reinforced with plant fiber-based nanofibers. Appl. Phys. A Mater. Sci. Process..

[97] Bhatnagar A., Sain M. (2005). Processing of Cellulose Nanofiber-reinforced Composites. J. Reinf. Plast. Compos..

[98] Wang S., Cheng Q. (2009). A novel process to isolate fibrils from cellulose fibers by high-intensity ultrasonication, Part 1: Process optimization. J. Appl. Polym. Sci..

[99] Janardhnan S., Sain M.M. (2006). Isolation of Cellulose Microfibrils—An Enzymatic Approach. Cellul. Microfibril Isol. Bioresour..

[100] Chen W., Yu H., Liu Y., Chen P., Zhang M., Hai Y. (2011). Individualization of cellulose nanofibers from wood using high-intensity ultrasonication combined with chemical pretreatments. Carbohydr. Polym..

[101] Qua E.H., Hornsby P.R., Sharma H.S.S., Lyons G. (2011). Preparation and characterisation of cellulose nanofibres. J. Mater. Sci..

[102] Zimmermann T., Pöhler E., Geiger T. (2004). Cellulose fibrils for polymer reinforcement. Adv. Eng. Mater..

[103] Qiu K., Netravali A.N. (2014). A Review of Fabrication and Applications of Bacterial Cellulose Based Nanocomposites. Polym. Rev..

[104] Chawla P.R., Bajaj I.B., Survase S.A., Singhal R.S. (2009). Microbial cellulose: Fermentative production and applications (Review). Food Technol. Biotechnol..

[105] Pircher N., Carbajal L., Schimper C., Bacher M., Rennhofer H., Nedelec J.M., Lichtenegger H.C., Rosenau T., Liebner F. (2016). Impact of selected solvent systems on the pore and solid structure of cellulose aerogels. Cellulose.

[106] Innerlohinger J., Weber H.K., Kraft G. (2006). Aerocellulose: Aerogels and aerogel-like materials made from cellulose. Macromol. Symp..

[107] Karadagli I., Schulz B., Schestakow M., Milow B., Gries T., Ratke L. (2015). Production of porous cellulose aerogel fibers by an extrusion process. J. Supercrit. Fluids.

[108] Jin H., Nishiyama Y., Wada M., Kuga S. (2004). Nanofibrillar cellulose aerogels. Colloids Surf. A Physicochem. Eng. Asp..

[109] Shi J., Lu L., Guo W., Liu M., Cao Y. (2015). On preparation, structure and performance of high porosity bulk cellulose aerogel. Plast. Rubber Compos..

[110] Cai J., Kimura S., Wada M., Kuga S., Zhang L. (2008). Cellulose aerogels from aqueous alkali hydroxide-urea solution. ChemSusChem.

[111] Schestakow M., Karadagli I., Ratke L. (2016). Cellulose aerogels prepared from an aqueous zinc chloride salt hydrate melt. Carbohydr. Polym..

[112] Chen C., Li J. (2011). Preparation of Lignocellulose Aerogel from Wood-Ionic Liquid Solution. Adv. Mater. Res..

[113] Duchemin B.J.C., Staiger M.P., Tucker N., Newman R.H. (2010). Aerocellulose based on all-cellulose composites. J. Appl. Polym. Sci..

[114] Chen M., Zhang X., Zhang A., Liu C., Sun R. (2016). Direct preparation of green and renewable aerogel materials from crude bagasse. Cellulose.

[115] Wang Z., Liu S., Matsumoto Y., Kuga S. (2012). Cellulose gel and aerogel from LiCl/DMSO solution. Cellulose.

[116] Hoepfner S., Ratke L., Milow B. (2008). Synthesis and characterisation of nanofibrillar cellulose aerogels. Cellulose.

[117] Li J., Lu Y., Yang D., Sun Q., Liu Y., Zhao H. (2011). Lignocellulose aerogel from wood-ionic liquid solution (1-allyl-3-methylimidazolium chloride) under freezing and thawing conditions. Biomacromolecules.

[118] Lu Y., Sun Q., Yang D., She X., Yao X., Zhu G., Liu Y., Zhao H., Li J. (2012). Fabrication of mesoporous lignocellulose aerogels from wood via cyclic liquid nitrogen freezing–thawing in ionic liquid solution. J. Mater. Chem..

[119] Liebner F., Potthast A., Rosenau T., Haimer E., Wendland M. (2008). Cellulose aerogels: Highly porous, ultra-lightweight materials. Holzforschung.

[120] Cai J., Kimura S., Wada M., Kuga S. (2009). Nanoporous cellulose as metal nanoparticles support. Biomacromolecules.

[121] Bao M.X., Xu S., Wang X., Sun R. (2016). Porous cellulose aerogels with high mechanical performance and their absorption behaviors. BioResources.

[122] Liao Q., Su X., Zhu W., Hua W., Qian Z., Liu L., Yao J. (2016). Flexible and durable cellulose aerogels for highly effective oil/water separation. RSC Adv..

[123] Fan P., Yuan Y., Ren J., Yuan B., He Q., Xia G., Chen F., Song R. (2017). Facile and green fabrication of cellulosed based aerogels for lampblack filtration from waste newspaper. Carbohydr. Polym..

[124] Nguyen S.T., Feng J., Ng S.K., Wong J.P.W., Tan V.B.C., Duong H.M. (2014). Advanced thermal insulation and absorption properties of recycled cellulose aerogels. Colloids Surf. A Physicochem. Eng. Asp..

[125] Jin C., Han S., Li J., Sun Q. (2015). Fabrication of cellulose-based aerogels from waste newspaper without any pretreatment and their use for absorbents. Carbohydr. Polym..

[126] Nguyen S.T., Feng J., Le N.T., Le A.T.T., Hoang N., Tan V.B.C., Duong H.M. (2013). Cellulose aerogel from paper waste for crude oil spill cleaning. Ind. Eng. Chem. Res..

[127] Lin R., Li A., Zheng T., Lu L., Cao Y. (2015). Hydrophobic and flexible cellulose aerogel as an efficient, green and reusable oil sorbent. RSC Adv..

[128] Zhang H., Li Y., Xu Y., Lu Z., Chen L., Huang L., Fan M. (2016). Versatile fabrication of a superhydrophobic and ultralight cellulose-based aerogel for oil spillage clean-up. Phys. Chem. Chem. Phys..

[129] Jian L.I., Caichao W.A.N., Yun L.U., Qingfeng S.U.N. (2014). Fabrication of cellulose aerogel from wheat straw with strong absorptive capacity. Front. Agric. Sci. Eng..

[130] Wan C., Lu Y., Cao J., Sun Q., Li J. (2015). Preparation, characterization and oil adsorption properties of cellulose aerogels from four kinds of plant materials via a NAOH/PEG aqueous solution. Fibers Polym..

[131] Cai J., Liu S., Feng J., Kimura S., Wada M., Kuga S., Zhang L. (2012). Cellulose-silica nanocomposite aerogels by in-situ formation of silica in cellulose gel. Angew. Chem. Int. Ed..

[132] Demilecamps A., Beauger C., Hildenbrand C., Rigacci A., Budtova T. (2015). Cellulose-silica aerogels. Carbohydr. Polym..

[133] Laskowski J., Milow B., Ratke L. (2015). The effect of embedding highly insulating granular aerogel in cellulosic aerogel. J. Supercrit. Fluids.

[134] Aaltonen O., Jauhiainen O. (2009). The preparation of lignocellulosic aerogels from ionic liquid solutions. Carbohydr. Polym..

[135] Lindman B., Karlström G., Stigsson L. (2010). On the mechanism of dissolution of cellulose. J. Mol. Liq..

[136] Medronho B., Lindman B. (2015). Brief overview on cellulose dissolution/regeneration interactions and mechanisms. Adv. Colloid Interface Sci..

[137] Medronho B., Lindman B. (2014). Competing forces during cellulose dissolution: From solvents to mechanisms. Curr. Opin. Colloid Interface Sci..

[138] Xiong B., Zhao P., Hu K., Zhang L., Cheng G. (2014). Dissolution of cellulose in aqueous NaOH/urea solution: Role of urea. Cellulose.

[139] Yan L., Gao Z. (2008). Dissolving of cellulose in PEG/NaOH aqueous solution. Cellulose.

[140] Zhang L., Ruan D., Gao S. (2002). Dissolution and regeneration of cellulose in NaOH/Thiourea aqueous solution. J. Polym. Sci. Part B Polym. Phys..

[141] Wang Z.G., Yokoyama T., Matsumoto Y. (2010). Dissolution of Ethylenediamine Pretreated Pulp with High Lignin Content in LiCl/DMSO without Milling. J. Wood Chem. Technol..

[142] Wang Z., Yokoyamaj T., Chang H.M., Matsumoto Y. (2009). Dissolution of beech and spruce milled woods in LiCI/DMSO. J. Agric. Food Chem..

[143] Ishii D., Tatsumi D., Matsumoto T., Murata K., Hayashi H., Yoshitani H. (2006). Investigation of the structure of cellulose in LiCl/DMAc solution and its gelation behavior by small-angle X-ray scattering measurements. Macromol. Biosci..

[144] Nishio Y., Hirose N. (1992). Cellulose/poly(2-hydroxyethyl methacrylate) composites prepared via solution coagulation and subsequent bulk polymerization. Polymer (Guildf.).

[145] Zhu S., Wu Y., Chen Q., Yu Z., Wang C., Jin S., Ding Y., Wu G. (2006). Dissolution of cellulose with ionic liquids and its application: A mini-review. Green Chem..

[146] EI-Wakil N.A., Hassan M.L. (2008). Structural Changes of Regenerated Cellulose Dissolved in FeTNa, NaOH/thiourea, and NMMO Systems. J. Appl. Polym. Sci..

[147] Fang Y., Chen S., Luo X., Wang C., Yang R., Zhang Q., Huang C., Shao T. (2016). Synthesis and characterization of cellulose triacetate aerogels with ultralow densities. RSC Adv..

[148] Fischer F., Rigacci A., Pirard R., Berthon-Fabry S., Achard P. (2006). Cellulose-based aerogels. Polymer.

[149] Yu M., Li J., Wang L. (2017). KOH-activated carbon aerogels derived from sodium carboxymethyl cellulose for high-performance supercapacitors and dye adsorption. Chem. Eng. J..

[150] Surapolchai W., Schiraldi D.A. (2010). The effects of physical and chemical interactions in the formation of cellulose aerogels. Polym. Bull..

[151] Martins B.F., de Toledo P.V.O., Petri D.F.S. (2017). Hydroxypropyl methylcellulose based aerogels: Synthesis, characterization and application as adsorbents for wastewater pollutants. Carbohydr. Polym..

[152] Kim C.H., Youn H.J., Lee H.L. (2015). Preparation of cross-linked cellulose nanofibril aerogel with water absorbency and shape recovery. Cellulose.

[153] Yang H., Sheikhi A., Van De Ven T.G.M. (2016). Reusable Green Aerogels from Cross-Linked Hairy Nanocrystalline Cellulose and Modified Chitosan for Dye Removal. Langmuir.

[154] Guilminot E., Fischer F., Chatenet M., Rigacci A., Berthon-Fabry S., Achard P., Chainet E. (2007). Use of cellulose-based carbon aerogels as catalyst support for PEM fuel cell electrodes: Electrochemical characterization. J. Power Sources.

[155] Choy Y.B., Choi H., Kim K. (2009). Uniform Ethyl Cellulose Microspheres of Controlled Sizes and Polymer Viscosities and Their Drug-Release Profiles. J. Appl. Polym. Sci..

[156] Chen B., Zheng Q., Zhu J., Li J., Cai Z., Chen L., Gong S. (2016). Mechanically strong fully biobased anisotropic cellulose aerogels. RSC Adv..

[157] Jiang F., Hsieh Y.-L. (2014). Super water absorbing and shape memory nanocellulose aerogels from TEMPO-oxidized cellulose nanofibrils via cyclic freezing–thawing. J. Mater. Chem. A.

[158] Sehaqui H., Zhou Q., Berglund L.A. (2011). High-porosity aerogels of high specific surface area prepared from nanofibrillated cellulose (NFC). Compos. Sci. Technol..

[159] Nemoto J., Saito T., Isogai A. (2015). Simple Freeze-Drying Procedure for Producing Nanocellulose Aerogel-Containing, High-Performance Air Filters. ACS Appl. Mater. Interfaces.

[160] Jiang F., Hsieh Y. (2017). Lo Cellulose nanofibril aerogels: Synergistic improvement of hydrophobicity, strength, and thermal stability via cross-linking with diisocyanate. ACS Appl. Mater. Interfaces.

[161] Zhai T., Zheng Q., Cai Z., Xia H., Gong S. (2016). Synthesis of polyvinyl alcohol/cellulose nanofibril hybrid aerogel microspheres and their use as oil/solvent superabsorbents. Carbohydr. Polym..

[162] Jiang F., Hsieh Y.-L. (2014). Amphiphilic superabsorbent cellulose nanofibril aerogels. J. Mater. Chem. A.

[163] Mulyadi A., Zhang Z., Deng Y. (2016). Fluorine-Free Oil Absorbents Made from Cellulose Nanofibril Aerogels. ACS Appl. Mater. Interfaces.

[164] Panzella L., Melone L., Pezzella A., Rossi B., Pastori N., Perfetti M., D’Errico G., Punta C., D’Ischia M. (2016). Surface-Functionalization of Nanostructured Cellulose Aerogels by Solid State Eumelanin Coating. Biomacromolecules.

[165] Buchtová N., Budtova T. (2016). Cellulose aero-, cryo- and xerogels: Towards understanding of morphology control. Cellulose.

[166] Sescousse R., Gavillon R., Budtova T. (2011). Aerocellulose from cellulose-ionic liquid solutions: Preparation, properties and comparison with cellulose-NaOH and cellulose-NMMO routes. Carbohydr. Polym..

[167] Pons A., Casas L., Estop E., Molins E., Harris K.D.M., Xu M. (2012). A new route to aerogels: Monolithic silica cryogels. J. Non-Cryst. Solids.

[168] Nakagaito A., Kondo H., Takagi H. (2013). Cellulose nanofiber aerogel production and applications. J. Reinf. Plast. Compos..

[169] Jiménez-Saelices C., Seantier B., Cathala B., Grohens Y. (2017). Spray freeze-dried nanofibrillated cellulose aerogels with thermal superinsulating properties. Carbohydr. Polym..

[170] Beaumont M., Kondor A., Plappert S., Mitterer C., Opietnik M., Potthast A., Rosenau T. (2016). Surface properties and porosity of highly porous, nanostructured cellulose II particles. Cellulose.

[171] Toyoda M., Inagaki M. (2003). Sorption and recovery of heavy oils by using exfoliated graphite. Spill Sci. Technol. Bull..

[172] Teas C., Kalligeros S., Zanikos F., Stournas S., Lois E., Anastopoulos G. (2001). Investigation of the effectiveness of absorbent materials in oil spills clean up. Desalination.

[173] Adebajo M.O., Frost R.L., Kloprogge J.T., Carmody O., Kokot S. (2003). Porous Materials for Oil Spill Cleanup: A Review of Synthesis. J. Porous Mater..

[174] Wang J., Zheng Y., Wang A. (2012). Effect of kapok fiber treated with various solvents on oil absorbency. Ind. Crops Prod..

[175] Ali N., El-Harbawi M., Jabal A.A., Yin C.Y. (2012). Characteristics and oil sorption effectiveness of kapok fibre, sugarcane bagasse and rice husks: Oil removal suitability matrix. Environ. Technol..

[176] Hussein M., Amer A.A., Sawsan I.I. (2011). Heavy oil spill cleanup using law grade raw cotton fibers: Trial for practical application. J. Pet. Technol..

[177] Khan E., Virojnagud W., Ratpukdi T. (2004). Use of biomass sorbents for oil removal from gas station runoff. Chemosphere.

[178] Likon M., Remškar M., Ducman V., Švegl F. (2013). Populus seed fibers as a natural source for production of oil super absorbents. J. Environ. Manag..

[179] Kaya M. (2017). Super absorbent, light, and highly flame retardant cellulose-based aerogel crosslinked with citric acid. J. Appl. Polym. Sci..

[180] Yang X., Cranston E.D. (2014). Chemically cross-linked cellulose nanocrystal aerogels with shape recovery and superabsorbent properties. Chem. Mater..

[181] Chong K.Y., Chia C.H., Zakaria S., Sajab M.S., Chook S.W., Khiew P.S. (2015). CaCO_3_-decorated cellulose aerogel for removal of Congo Red from aqueous solution. Cellulose.

[182] Wei X., Huang T., Yang J.H., Zhang N., Wang Y., Zhou Z.W. (2017). Green synthesis of hybrid graphene oxide/microcrystalline cellulose aerogels and their use as superabsorbents. J. Hazard. Mater..

[183] Feng J., Nguyen S.T., Fan Z., Duong H.M. (2015). Advanced fabrication and oil absorption properties of super-hydrophobic recycled cellulose aerogels. Chem. Eng. J..

[184] Cheng H., Gu B., Pennefather M.P., Nguyen T.X., Phan-Thien N., Duong H.M. (2017). Cotton aerogels and cotton-cellulose aerogels from environmental waste for oil spillage cleanup. Mater. Des..

[185] Mi H.Y., Jing X., Politowicz A.L., Chen E., Huang H.X., Turng L.S. (2018). Highly compressible ultra-light anisotropic cellulose/graphene aerogel fabricated by bidirectional freeze drying for selective oil absorption. Carbon N. Y..

[186] Fumagalli M., Ouhab D., Boisseau S.M., Heux L. (2013). Versatile gas-phase reactions for surface to bulk esterification of cellulose microfibrils aerogels. Biomacromolecules.

[187] Granström M., née Pääkkö M.K., Jin H., Kolehmainen E., Kilpeläinen I., Ikkala O. (2011). Highly water repellent aerogels based on cellulose stearoyl esters. Polym. Chem..

[188] Russler A., Wieland M., Bacher M., Henniges U., Miethe P., Liebner F., Potthast A., Rosenau T. (2012). AKD-Modification of bacterial cellulose aerogels in supercritical CO_2_. Cellulose.

[189] Ge J., Zhao H.Y., Zhu H.W., Huang J., Shi L.A., Yu S.H. (2016). Advanced Sorbents for Oil-Spill Cleanup: Recent Advances and Future Perspectives. Adv. Mater..

[190] Ma Q., Cheng H., Fane A.G., Wang R., Zhang H. (2016). Recent Development of Advanced Materials with Special Wettability for Selective Oil/Water Separation. Small.

[191] Peng H., Wu J., Wang Y., Wang H., Liu Z., Shi Y., Guo X. (2016). A facile approach for preparation of underwater superoleophobicity cellulose/chitosan composite aerogel for oil/water separation. Appl. Phys. A Mater. Sci. Process..

[192] Sai H., Xing L., Xiang J., Cui L., Jiao J., Zhao C., Li Z., Li F. (2013). Flexible aerogels based on an interpenetrating network of bacterial cellulose and silica by a non-supercritical drying process. J. Mater. Chem. A.

[193] Pierre A.C., Pajonk G.M. (2002). Chemistry of aerogels and their applications. Chem. Rev..

[194] Koebel M., Rigacci A., Achard P. (2012). Aerogel-based thermal superinsulation: An overview. J. Sol-Gel Sci. Technol..

[195] Sequeira S., Evtuguin D.V., Portugal I. (2009). Preparation and properties of cellulose/silica hybrid composites. Polym. Compos..

[196] Sedighi Gilani M., Boone M.N., Fife J.L., Zhao S., Koebel M.M., Zimmermann T., Tingaut P. (2016). Structure of cellulose-silica hybrid aerogel at sub-micron scale, studied by synchrotron X-ray tomographic microscopy. Compos. Sci. Technol..

[197] Fu J., He C., Wang S., Chen Y. (2018). A thermally stable and hydrophobic composite aerogel made from cellulose nanofibril aerogel impregnated with silica particles. J. Mater. Sci..

[198] Zhou S., Zhou L., Li Y., Xie F., Li H., Yang H., Li W., Snyders R. (2018). Preparation of cellulose-graphene oxide aerogels with N-methyl morpholine-N-oxide as a solvent. J. Appl. Polym. Sci..

[199] Hu P., Tan B., Long M. (2016). Advanced nanoarchitectures of carbon aerogels for multifunctional environmental applications. Nanotechnol. Rev..

[200] Thamilselvan A., Nesaraj A.S., Noel M. (2016). Review on carbon-based electrode materials for application in capacitive deionization process. Int. J. Environ. Sci. Technol..

[201] Du A., Zhou B., Zhang Z., Shen J. (2013). A special material or a new state of matter: A review and reconsideration of the aerogel. Materials.

[202] Lee Y.J., Jung J.C., Yi J., Baeck S.H., Yoon J.R., Song I.K. (2010). Preparation of carbon aerogel in ambient conditions for electrical double-layer capacitor. Curr. Appl. Phys..

[203] Wan C., Lu Y., Jiao Y., Jin C., Sun Q., Li J. (2015). Fabrication of hydrophobic, electrically conductive and flame-resistant carbon aerogels by pyrolysis of regenerated cellulose aerogels. Carbohydr. Polym..

[204] Jiao Y., Wan C., Li J. (2016). Synthesis of carbon fiber aerogel from natural bamboo fiber and its application as a green high-efficiency and recyclable adsorbent. Mater. Des..

[205] White R.J., Brun N., Budarin V.L., Clark J.H., Titirici M.M. (2014). Always look on the “light” side of life: Sustainable carbon aerogels. ChemSusChem.

[206] Zhuo H., Hu Y., Tong X., Zhong L., Peng X., Sun R. (2016). Sustainable hierarchical porous carbon aerogel from cellulose for high-performance supercapacitor and CO_2_ capture. Ind. Crops Prod..

[207] Hu Y.J., Tong X., Zhuo H., Zhong L.X., Peng X.W., Wang S., Sun R.C. (2016). 3D hierarchical porous N-doped carbon aerogel from renewable cellulose: An attractive carbon for high-performance supercapacitor electrodes and CO_2_ adsorption. RSC Adv..

[208] Wan C., Li J. (2016). Graphene oxide/cellulose aerogels nanocomposite: Preparation, pyrolysis, and application for electromagnetic interference shielding. Carbohydr. Polym..

[209] Wan C., Jiao Y., Qiang T., Li J. (2017). Cellulose-derived carbon aerogels supported goethite (α-FeOOH) nanoneedles and nanoflowers for electromagnetic interference shielding. Carbohydr. Polym..

[210] Chen Z., Peng X., Zhang X., Jing S., Zhong L., Sun R. (2017). Facile synthesis of cellulose-based carbon with tunable N content for potential supercapacitor application. Carbohydr. Polym..

[211] Yu M., Han Y., Li J., Wang L. (2017). One-step synthesis of sodium carboxymethyl cellulose-derived carbon aerogel/nickel oxide composites for energy storage. Chem. Eng. J..

[212] Li C., Wu Z.Y., Liang H.W., Chen J.F., Yu S.H. (2017). Ultralight Multifunctional Carbon-Based Aerogels by Combining Graphene Oxide and Bacterial Cellulose. Small.

[213] Yu M., Li J., Wang L. (2016). Preparation and characterization of magnetic carbon aerogel from pyrolysis of sodium carboxymethyl cellulose aerogel crosslinked by iron trichloride. J. Porous Mater..

[214] Yu M., Han Y., Li J., Wang L. (2017). Magnetic carbon aerogel pyrolysis from sodium carboxymethyl cellulose/sodium montmorillonite composite aerogel for removal of organic contamination. J. Porous Mater..

[215] Tian X., Zhu S., Peng J., Zuo Y., Wang G., Guo X., Zhao N., Ma Y., Ma L. (2017). Synthesis of micro- and meso-porous carbon derived from cellulose as an electrode material for supercapacitors. Electrochim. Acta.

[216] Yang X., Fei B., Ma J., Liu X., Yang S., Tian G., Jiang Z. (2018). Porous nanoplatelets wrapped carbon aerogels by pyrolysis of regenerated bamboo cellulose aerogels as supercapacitor electrodes. Carbohydr. Polym..

[217] Wang Y., Wang Y., Jiang L. (2018). Freestanding carbon aerogels produced from bacterial cellulose and its Ni/MnO_2_/Ni(OH)_2_ decoration for supercapacitor electrodes. J. Appl. Electrochem..

[218] Zhang S., Feng J., Feng J., Jiang Y., Ding F. (2018). Carbon aerogels by pyrolysis of TEMPO-oxidized cellulose. Appl. Surf. Sci..

[219] Zhang C., Zhai T., Turng L.S. (2017). Aerogel microspheres based on cellulose nanofibrils as potential cell culture scaffolds. Cellulose.

[220] Henschen J., Illergård J., Larsson P.A., Ek M., Wågberg L. (2016). Contact-active antibacterial aerogels from cellulose nanofibrils. Colloids Surf. B Biointerfaces.

[221] Uddin K.M.A., Orelma H., Mohammadi P., Borghei M., Laine J., Linder M., Rojas O.J. (2017). Retention of lysozyme activity by physical immobilization in nanocellulose aerogels and antibacterial effects. Cellulose.

[222] Abu-Izza K., Tambrallo L., Lu D.R. (1997). In Vivo Evaluation of Zidovudine (AZT)-Loaded Ethylcellulose Microspheres after Oral Administration in Beagle Dogs. Society.

[223] Shen Z., Mitragotri S. (2002). Intestinal patches for oral drug delivery. Pharm. Res..

[224] Zhao J., Lu C., He X., Zhang X., Zhang W., Zhang X. (2015). Polyethylenimine-grafted cellulose nanofibril aerogels as versatile vehicles for drug delivery. ACS Appl. Mater. Interfaces.

[225] Wang R., Shou D., Lv O., Kong Y., Deng L., Shen J. (2017). pH-Controlled drug delivery with hybrid aerogel of chitosan, carboxymethyl cellulose and graphene oxide as the carrier. Int. J. Biol. Macromol..

[226] Vincent Edwards J., Fontenot K.R., Prevost N.T., Pircher N., Liebner F., Condon B.D. (2016). Preparation, characterization and activity of a peptide-cellulosic aerogel protease sensor from cotton. Sensors.

[227] Park J.E., Kim K., Jung Y., Kim J.H., Nam J.M. (2016). Metal Nanoparticles for Virus Detection. ChemNanoMat.

[228] Crooks R.M., Zhao M., Sun L., Chechik V., Yeung L.K. (2001). Dendrimer-encapsulated metal nanoparticles: Synthesis, characterization, and applications to catalysis. Acc. Chem. Res..

[229] Kamat P.V. (2002). Photophysical, photochemical and photocatalytic aspects of metal nanoparticles. J. Phys. Chem. B.

[230] Luong N.D., Lee Y., Nam J. (2008). Do Highly-loaded silver nanoparticles in ultrafine cellulose acetate nanofibrillar aerogel. Eur. Polym. J..

[231] Schestakow M., Muench F., Reimuth C., Ratke L., Ensinger W. (2016). Electroless synthesis of cellulose-metal aerogel composites. Appl. Phys. Lett..

[232] Keshipour S., Khezerloo M. (2017). Gold nanoparticles supported on cellulose aerogel as a new efficient catalyst for epoxidation of styrene. J. Iran. Chem. Soc..

[233] Keshipour S., Khezerloo M. (2018). Au-dimercaprol functionalized cellulose aerogel: Synthesis, characterization and catalytic application. Appl. Organomet. Chem..

[234] Thiruvengadam V., Vitta S. (2016). Interparticle interactions mediated superspin glass to superferromagnetic transition in Ni-bacterial cellulose aerogel nanocomposites. J. Appl. Phys..

[235] Yao Q., Fan B., Xiong Y., Wang C., Wang H., Jin C., Sun Q. (2017). Stress sensitive electricity based on Ag/cellulose nanofiber aerogel for self-reporting. Carbohydr. Polym..

[236] Zhang X., Lin Z., Chen B., Zhang W., Sharma S., Gu W., Deng Y. (2014). Solid-state flexible polyaniline/silver cellulose nanofibrils aerogel supercapacitors. J. Power Sources.

[237] Kettunen M., Silvennoinen R.J., Houbenov N., Nykänen A., Ruokolainen J., Sainio J., Pore V., Kemell M., Ankerfors M., Lindström T. (2011). Photoswitchable superabsorbency based on nanocellulose aerogels. Adv. Funct. Mater..

[238] Korhonen J.T., Kettunen M., Ras R.H.A., Ikkala O. (2011). Hydrophobic nanocellulose aerogels as floating, sustainable, reusable, and recyclable oil absorbents. ACS Appl. Mater. Interfaces.

[239] Liu Z., Wu P., Yang S., Wang H., Jin C. (2016). Synthesis and Characterization of Uniform Spherical Nanoporous TiO_2_ Aerogel Templated by Cellulose Alcohol-Gel with Enhanced Photocatalytic Activity. Int. J. Polym. Sci..

[240] Zhao H.B., Zhou X.C., Fu Z.B., Mi R., Wang C.Y. (2017). Freestanding monolithic Ni aerogel with large surface areas from cellulose aerogel templates. Mater. Lett..

[241] Liao H., Zhang H., Hong H., Li Z., Qin G., Zhu H., Lin Y. (2016). Novel cellulose aerogel coated on polypropylene separators as gel polymer electrolyte with high ionic conductivity for lithium-ion batteries. J. Membr. Sci..

[242] Yang J., Zhang E., Li X., Zhang Y., Qu J., Yu Z.Z. (2016). Cellulose/graphene aerogel supported phase change composites with high thermal conductivity and good shape stability for thermal energy storage. Carbon N. Y..

[243] Qin J., Chen L., Zhao C., Lin Q., Chen S. (2017). Cellulose nanofiber/cationic conjugated polymer hybrid aerogel sensor for nitroaromatic vapors detection. J. Mater. Sci..

[244] Kobayashi Y., Saito T., Isogai A. (2014). Angewandte Aerogels with 3D Ordered Nanofiber Skeletons of Liquid-Crystalline Nanocellulose Derivatives as Tough and Transparent Insulators **. Angew. Chem. Int. Ed..

